# Drought tolerance in selected aerobic and upland rice varieties is driven by different metabolic and antioxidative responses

**DOI:** 10.1007/s00425-021-03659-4

**Published:** 2021-06-25

**Authors:** Giovanni Melandri, Hamada AbdElgawad, Kristýna Floková, Diaan C. Jamar, Han Asard, Gerrit T. S. Beemster, Carolien Ruyter-Spira, Harro J. Bouwmeester

**Affiliations:** 1 Laboratory of Plant Physiology, Wageningen University and Research, Wageningen, The Netherlands; 2 Laboratory for Integrated Molecular Plant Physiology Research (IMPRES), University of Antwerp, Antwerp, Belgium; 3 Department of Botany, Faculty of Science, Beni-Suef University, Beni Suef, Egypt; 4 Plant Hormone Biology Group, Swammerdam Institute for Life Sciences, University of Amsterdam, Amsterdam, the Netherlands; 5 Laboratory of Growth Regulators, Centre of the Region Haná for Biotechnological and Agricultural Research, Institute of Experimental Botany AS CR and Palacký University, Olomouc, Czech Republic; 6 Present Address: School of Plant Sciences, The University of Arizona, Tucson, AZ USA

**Keywords:** Antioxidative response, Biomass, Drought, Metabolism, *Oryza sativa*, Osmotic adjustment, Vegetative stage

## Abstract

**Main conclusions:**

Sugar-mediated osmotic acclimation and a strong antioxidative response reduce drought-induced biomass loss at the vegetative stage in rice.

**Abstract:**

A clear understanding of the physiological and biochemical adaptations to water limitation in upland and aerobic rice can help to identify the mechanisms underlying their tolerance to low water availability. In this study, three *indica* rice varieties-IR64 (lowland), Apo (aerobic), and UPL Ri-7 (upland)-, that are characterized by contrasting levels of drought tolerance, were exposed to drought at the vegetative stage. Drought-induced changes in biomass, leaf metabolites and oxidative stress markers/enzyme activities were analyzed in each variety at multiple time points. The two drought-tolerant varieties, Apo and UPL Ri-7 displayed a reduced water use in contrast to the susceptible variety IR64 that displayed high water consumption and consequent strong leaf dehydration upon drought treatment. A sugar-mediated osmotic acclimation in UPL Ri-7 and a strong antioxidative response in Apo were both effective in limiting the drought-induced biomass loss in these two varieties, while biomass loss was high in IR64, also after recovery. A qualitative comparison of these results with the ones of a similar experiment conducted in the field at the reproductive stage showed that only Apo, which also in this stage showed the highest antioxidant power, was able to maintain a stable grain yield under stress. Our results show that different metabolic and antioxidant adaptations confer drought tolerance to aerobic and upland rice varieties in the vegetative stage. The effectiveness of these adaptations differs between developmental stages. Unraveling the genetic control of these mechanisms might be exploited in breeding for new rice varieties adapted to water-limited environments.

**Supplementary Information:**

The online version contains supplementary material available at 10.1007/s00425-021-03659-4.

## Introduction

In the coming decades, drought episodes associated with global climate change are projected to become more frequent and erratic. Leng and Hall (2019) estimated that by the end of the twenty-first century, the risk of drought-induced grain yield loss in the major Asian rice producing countries will increase by 18–19% compared to present conditions. In this context, improving drought tolerance in rice will be critical to meet the growing global food demand, particularly considering that, in Asia, ~ 40% of the total crop area is cultivated in rainfed agroecosystems (FAO 2014) which are prone to droughts.

Drought induces changes in the morphology and physiology of plants that ultimately result in metabolic reprogramming to adapt to the stress. The extent of this reprogramming is key in the trade-off between growth and survival. Essential metabolism under stressful conditions needs to be maintained, while anti-stress agents such as compatible solutes, antioxidants, stress-responsive proteins and enzymes need to be produced (Obata and Fernie 2012; Claeys and Inze 2013). Growth reduction is an early response to water limitation and frequently occurs without any alteration in photosynthetic rate (Skirycz and Inzé 2010; Fàbregas and Fernie 2019). Prolonged drought induces stomatal closure to reduce water loss, but this also limits photosynthetic CO_2_ assimilation, resulting in metabolic alterations and constraints (Chaves et al. 2009; Pinheiro and Chaves 2011). As a consequence, adjusting carbohydrate biosynthesis and translocation, for example, for osmoregulation, plays a central role in the response to drought (Luquet et al. 2008; Hummel et al. 2010; Muller et al. 2011). Previous research on drought, has shown that accumulation of particular metabolites in leaves (e.g., raffinose, trehalose, proline, and glycine betaine) can have a protective function whereas the accumulation of other metabolites may simply be a consequence of drought (for example the increase in free amino acids from protein breakdown) (Verslues and Juenger 2011; Krasensky and Jonak 2012; Obata and Fernie 2012; Fàbregas and Fernie 2019). Another effect of drought-induced stomatal closure and lower carbon availability, is an enhanced generation of reactive oxygen species (ROS) (Suzuki et al. 2012; Noctor et al. 2014) responsible for oxidative damage that drives the cell into senescence (Halliwell 2006) and, in extreme cases, death (Van Breusegem and Dat 2006). A complex enzymatic and non-enzymatic antioxidative system protects plants against this oxidative damage, and is essential for conferring drought tolerance (Mittler et al. 2011; Baxter et al. 2014; You and Chan 2015; Soares et al. 2019).

An additional layer of complexity in plant stress responses to drought relates to the fact that the stress has a different impact on plant performance at different developmental stages. For this reason, the reproductive stage is often targeted in drought experiments involving cereals, as the occurrence of the stress at this stage results in the most severe grain yield reduction (Passioura 2012; Biswal and Kohli 2013; Reynolds et al. 2016). This is particularly true for rice, in which even moderate stress during flowering can result in a strongly reduced grain yield (Liu et al. 2006; Venuprasad et al. 2007; Sandhu et al. 2014). Nevertheless, in the coming years, cultivation of rice under non-flooded conditions, in non-puddled and non-water-saturated soil (‘aerobic rice’) during the entire crop cycle is expected to expand (Venuprasad et al. 2012). Therefore, studies aimed at better understanding drought tolerance mechanisms in rice also at the vegetative stage will contribute to the selection of genotypes with better adaptation to water-limited conditions throughout the crop life cycle.

In this study, we investigated, in a growth chamber experiment under controlled conditions, how changes in leaf metabolism and oxidative stress status during vegetative development are associated with morphological and physiological changes (water consumption over time, biomass accumulation and leaf water status) during progressive drought and after re-watering. The study considered three rice varieties with contrasting levels of drought tolerance, (i) IR64, a lowland high-yielding variety that is commonly grown under flooded conditions and is highly susceptible to drought as indicated by its considerable reduction of grain yield when grown under aerobic conditions (Mackill and Khush 2018); (ii) Apo, a moderately drought-tolerant aerobic-adapted variety with good yield potential (Venuprasad et al. 2012); and (iii) UPL Ri-7, a drought-tolerant upland-adapted variety with improved yield potential (Atlin et al. 2006). The effect of drought on the leaf metabolic and oxidative stress profiles of these varieties during vegetative development was also compared with the profiles of the same varieties collected from a different study when drought was applied at the reproductive stage, under field conditions. This qualitative comparison allowed to get insight into similarities and differences between the drought coping strategies in the two different phenological stages.

## Materials and methods

### Growth chamber experiment at the vegetative stage

Seeds of the three *indica* rice (*Oryza sativa*) varieties were obtained from the International Rice Research Institute (IRRI) gene bank: (i) IR64 (IRGC 117268); (ii) Apo (IRGC 115128); (iii) UPL Ri-7 (IRTP 9897) (hereafter called Ri-7). Seeds were directly sown into 1.75 L pots (14 cm diameter, 18 cm height) filled with the same amount of dry clay loam soil. Plants (one per pot) were grown in a growth chamber (12-h photoperiod, 28/23 °C (d/n); 75/70% relative humidity (d/n); 600 µmol m^−2^ s^−1^ PAR) and watered (100% of maximum soil moisture content based on fully drained weight) every second day with ½ strength Hoagland’s solution until day 24 after sowing. On day 25 after sowing (vegetative stage, pre-tillering), all plants were watered to the same level of soil moisture (140% of maximum soil moisture content, flooded). Well-watered plants were kept flooded during the entire experiment duration. Plants exposed to drought were not watered for 10 consecutive days before being re-watered to flooded conditions (140% of maximum soil moisture content). Pots of drought-treated plants were covered with a reflecting disc (Suppl. Fig. S1) to reduce water evaporation from the soil. Pots were arranged on trolleys with adjustable heights to compensate for the different height of the three rice varieties and to expose all the plants to the same light intensity. For each rice variety and treatment, forty-eight plants were arranged in 2 trolleys each one comprising 24 pots (Suppl. Fig. S1). Trolleys were arranged in a randomized design and their position was rotated daily to minimize the effect of environmental differences inside the growth chamber. Every day, the weight of twelve drought-treated plants per variety was measured to determine transpiration. Four time points were selected for biomass measurements and leaf sampling: TP1, 4 days after start of stress; TP2, at around 40% of maximum soil moisture content, independently from the day after start of stress; TP3, 10 days after start of stress (severe drought); TP4, 2 days after re-watering (recovery time point). At each time point, for every genotype, 3 control and 3 stressed plants were randomly selected and the number of tillers, dry weight of leaves, tillers and roots, and the relative water content (RWC) of a fully developed leaf of a tiller were analyzed. RWC was expressed as (Fresh-Dry weight)/(Saturated-Dry weight)*100%. At each time point, towards the middle of the day (6 h of light), 12 plants of the same variety and treatment were randomly selected and sampled for metabolite analyses (and not sampled again in the other time points). The last two fully developed leaves on the primary tiller of 3 plants (of the 12) were collected, pooled and snap-frozen in liquid nitrogen to generate a biological replicate (4 biological replicates per variety, treatment, and time point). At TP4, necrotic leaf parts of drought-stressed plants were not considered for metabolite and oxidative stress analyses nor for biomass determination.

### Field experiment at the reproductive stage

The same three rice varieties were grown in a large field trial (~ 300 accessions) conducted at the International Rice Research Institute (IRRI), Philippines, during the 2013 dry season. The experiment comprised of a well-watered (flooded) field and a drought field, each including three replicated plots of the three rice varieties. Drought (14 consecutive days of water withholding, equal to − 46 kPa at 30 cm depth at the end of stress) was applied only to the stress field at the reproductive stage (50% flowering). At the end of the stress treatment, the field was re-watered until all the three varieties reached maturity for harvest (in approx. 30 days). Sampling of leaves from drought-treated plants was done on day 14 of the stress treatment; while for well-watered plants, the sampling was conducted two days later. From each replicated plot of the three varieties, eight flag leaves were sampled from the main tiller of eight different plants and snap-frozen in liquid nitrogen in the field. Further details on the management of the field trial and sampling can be found in Kadam et al. (2018) and Melandri et al. (2020).

### Analysis of primary metabolites

Leaf samples collected in the growth chamber experiment at the vegetative stage were analyzed for sugar (sucrose, fructose, glucose, trehalose and xylose) levels using a Dionex HPLC system according to Bentsink et al. (2000), with minor modifications. The same samples were analyzed for their levels of nutrient ions (phosphate, nitrate and sulfate) and organic acids (citrate, isocitrate and α-ketoglutarate) using a Dionex HPLC system as described by He et al. (2014), with minor modifications. The quantification of amino acids (Alanine: Ala; Serine: Ser; Proline: Pro; Valine: Val; Threonine: Thr; Isoleucine: Ile; Leucine: Leu; Aspartate: Asp; Glutamine: Glu; Methionine: Met; Histidine: His; Phenylalanine: Phe; Arginine: Arg; Tyrosine: Tyr; Lysine: Lys; Glycine: Gly; *gamma*-Aminobutyric acid: GABA; Asparagine: Asn; Glutamine: Gln; Tryptophan: Trp; Ornithine: Orn) in the same leaf samples was performed using a UPLC–MS/MS according to Carreno-Quintero et al. (2014) with modifications. Detailed protocols for the analysis of primary metabolites of the samples collected in the growth chamber experiment can be found in Suppl. Methods S1.

Flag leaf samples of the three rice varieties collected in the field experiment at the reproductive stage were analyzed using untargeted metabolite profiling by GC–MS, while the three main sugars (sucrose, fructose and glucose) were quantified spectrophotometrically. For each variety and treatment, equal amounts of leaf tissue from replicated plots (representing a total of 24 flag leaves from 24 different plants) were pooled together before the analyses. A more detailed description of the methods used for the biochemical analyses of the samples from the field experiment can be found in Melandri et al. (2020). Nutrient ions (phosphate, nitrate and sulfate) were not quantified in the leaf samples of the field experiment.

### Quantification of oxidative stress markers and antioxidant enzyme activities

Leaf samples of the growth chamber experiment and the field experiment were analyzed for their oxidative stress status. The lipid peroxidation product malondialdehyde (MDA) was assayed according to Hodges et al. (1999). Total antioxidant capacity (TAC) was assayed using ferric reducing antioxidant power (FRAP) reagent according to Benzie and Strain (1999). The amount of soluble protein in each sample was quantified by the Lowry method (Lowry et al. 1951). Protein carbonylation (ProtOx) was assayed according to Levine et al. (1994). Ascorbate peroxidase (APX), dehydroascorbate reductase (DHAR), superoxide dismutase (SOD) and catalase (CAT) activities were measured using a micro-plate reader assay (Dhindsa et al. 1982; Murshed et al. 2008; Sugawara and Nikaido 2014). Detailed protocols of the assays used to quantify oxidative stress markers and antioxidant enzyme activities can be found in Suppl. Methods S1.

### Statistical analyses

All statistical analyses were performed using R statistical software (version 3.5.1; The R Foundation for Statistical Computing). Analysis of variance (One-way ANOVA) and Tukey Honest Significant Differences (TukeyHSD) test were used to compare soil moisture content of each genotype at each time point, setting the threshold for statistical significance at *P* < 0.05. Student’s *t*-test was used to determine significant differences (*P* < 0.05) of growth-related traits under the two different treatments for each variety at each time point. Imputation of missing metabolic and oxidative stress values, prior to any other statistical test, was performed by the function *knnImputation* in the ‘DMwR’ R package. For each time point, two-factorial ANOVA (Two-way ANOVA) was performed to evaluate the significance (Bonferroni-corrected *P* value < 0.05) of the effect of treatment, variety and their interaction on the levels of metabolites, oxidative stress markers and antioxidant enzyme activities (log_10_ transformed before ANOVA to increase normality). For the growth chamber experiment, fold-change analysis was performed by dividing the mean value of each individual metabolic and oxidative stress marker/enzyme under drought by the mean value of the same metabolite and oxidative stress state marker/enzyme under control conditions for each variety at each time point. For the field experiment, fold-change analysis of the three varieties was performed by dividing the single value of each metabolic and oxidative stress marker/enzyme under drought by the relative single value under well-watered conditions. PCA was performed using the function *prcomp* in the ‘stats’ R package. Each metabolic and oxidative stress marker/enzyme value was log_10_ transformed to improve normality, centered (mean subtraction) and scaled (standard deviation division) before PCA.

## Results

### Plant transpiration and leaf water status

**Fig. 1:**
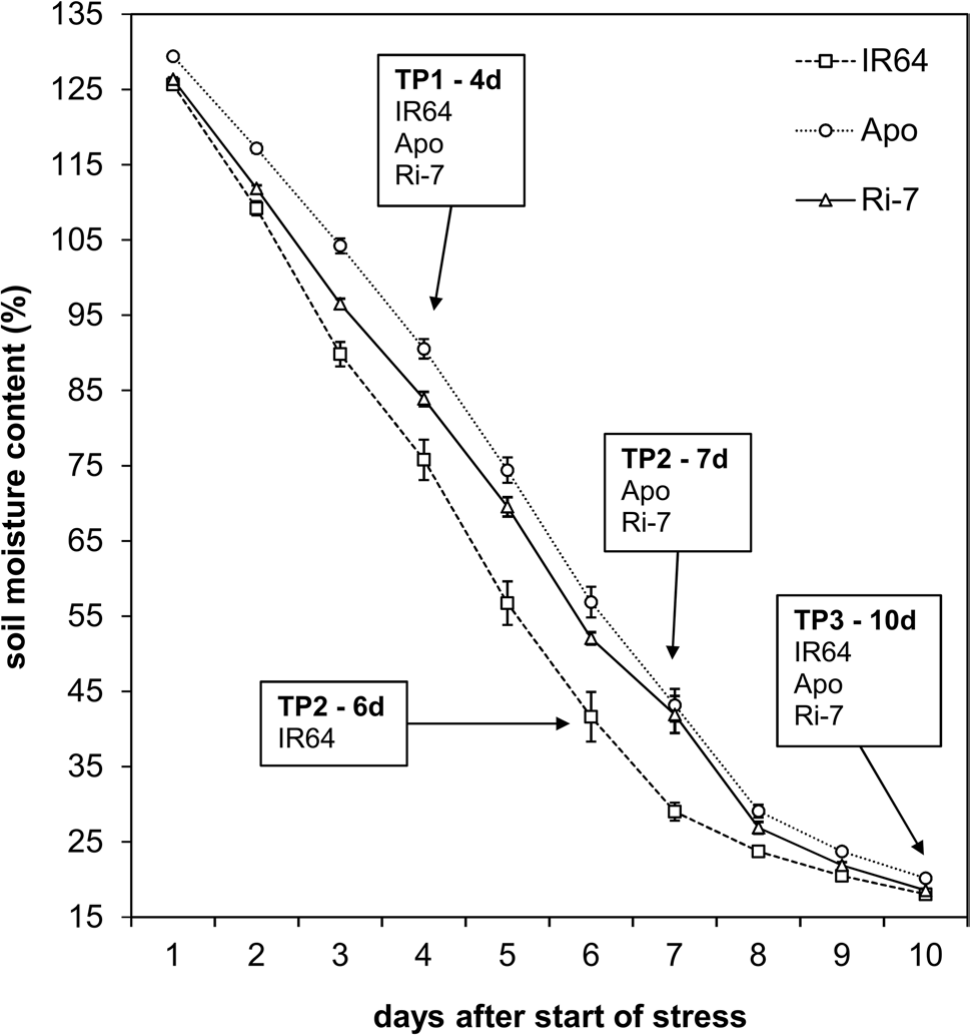
Evolution of soil water content in the three rice varieties. Each data point represents the mean value of soil moisture content (%) of 12 different plants (± SE) per variety. The first time point (TP1, early-mild drought) was selected 4 days after water withholding for all varieties. The second time point (TP2) was selected targeting a same specific soil moisture content (~ 40% of maximum soil moisture content) between the varieties (6 days after water withholding in IR64 and 7 days in Apo and Ri-7). The third time point (TP3) was selected 10 days after water withholding for all varieties

During the growth chamber experiment, the daily decrease in pot water content-expressed as percentage of maximum soil moisture content-was used as proxy for plant transpiration. At time point (TP) 1 (4 days after water withholding), pot water content was significantly different (*P* < 0.001) between the varieties, with IR64 showing the highest water consumption, followed by Ri-7 and Apo (Fig. 1 and Suppl. Fig. S2). TP2 was selected such that it represents the same soil moisture content (~ 40% of maximum soil moisture content) for all three varieties. This allowed a comparison of their physiological and biochemical responses at the same drought intensity (Suppl. Fig. S2). For IR64, 40% of maximum soil moisture content was reached one day earlier (6 days after water withholding) than for Apo and Ri-7 (7 days), confirming the higher water consumption under drought for the first variety (Fig. 1). At TP3 (10 days after water withholding), pot water contents were extremely low (~ 20% of maximum soil moisture content) for all varieties (Fig. 1) with, however, Apo displaying significantly (*P* < 0.001) less water depletion than IR64 and Ri-7 (Suppl. Fig. S2).

**Fig. 2:**
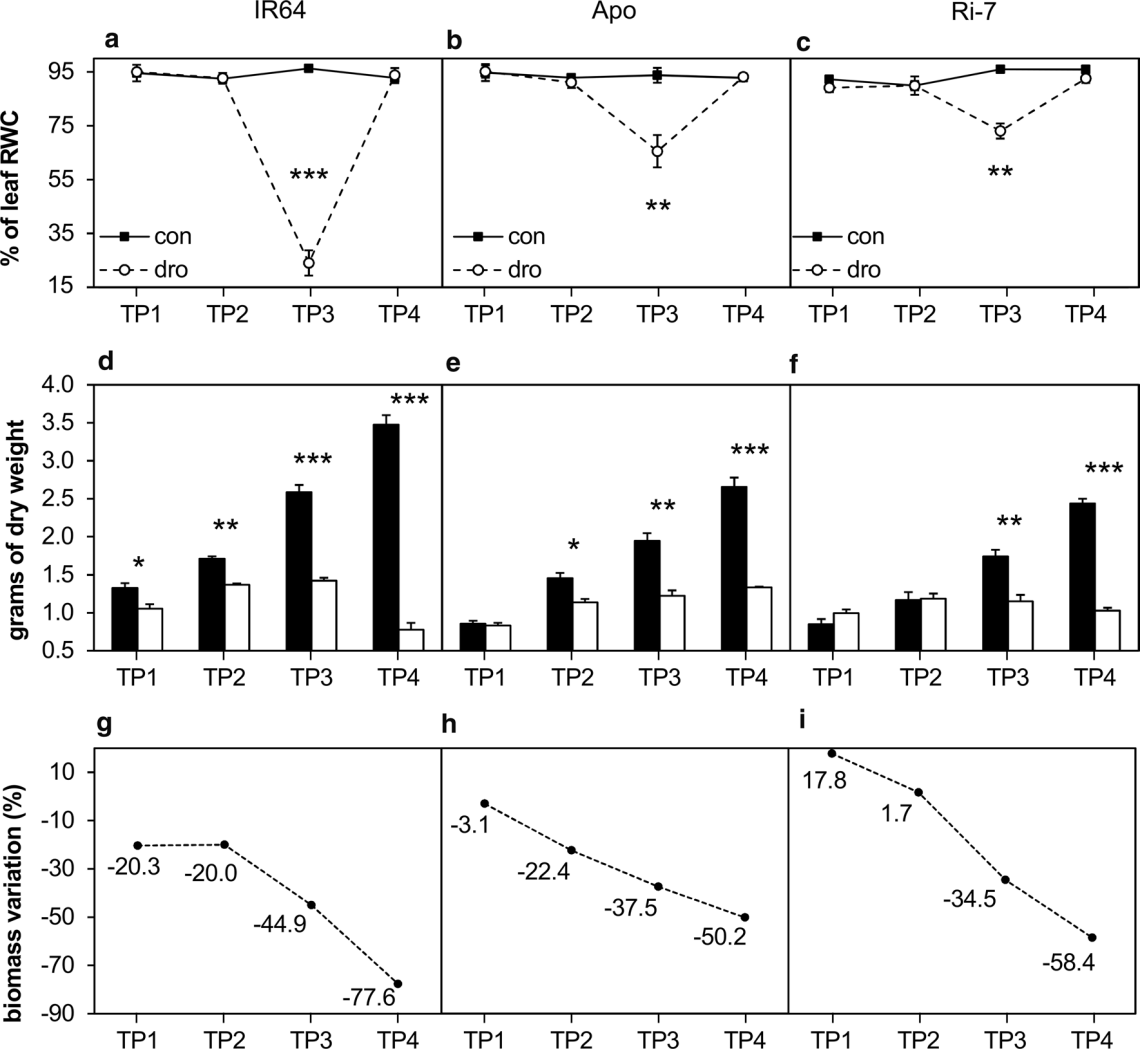
Evolution of leaf water content and biomass in response to drought in the three rice varieties. **a**–**c** The percentage of leaf relative water content (RWC) of the top fully developed leaf on the main tiller of plants exposed to control and drought conditions at each time point (TP1, TP2, TP3, TP4). **d**, **e,** and **f** Leaf biomass (dry weight) of the three varieties at the four time points. **g**, **h,** and **i** Leaf biomass difference (%) between control and drought plants at each time point. Black and white columns represent the mean ± standard error (SE) of three biological replicates of control and drought plants, respectively. Each data point represents the mean ± standard error (SE) of three biological replicates. ***, **, *Represent *t*-test’s *P* values of significance between control and drought replicates with *P* ≤ 0.001, *P* ≤ 0.01, *P* ≤ 0.05, respectively

To get insight into the progression of plant dehydration, the relative water content (RWC) of the youngest fully developed leaf on a main tiller was determined (Fig. 2a–c and Suppl. Table S1). Control plants of all varieties displayed a stable leaf RWC, higher than 90%, during all four TPs. Drought-treated plants only showed a significant difference in RWC from their controls at the end of the stress (TP3), with IR64 displaying a much lower value (24%) than the other two (~ 70%).

Collectively, these results suggest that the drought sensitivity of IR64 was fostered by a high and sustained water consumption resulting in strong leaf dehydration at the end of the stress period. In contrast, the more drought-tolerant varieties Apo and Ri-7 had a lower water consumption and suffered less dehydration.

### Growth responses to increasing drought

**Fig. 3:**
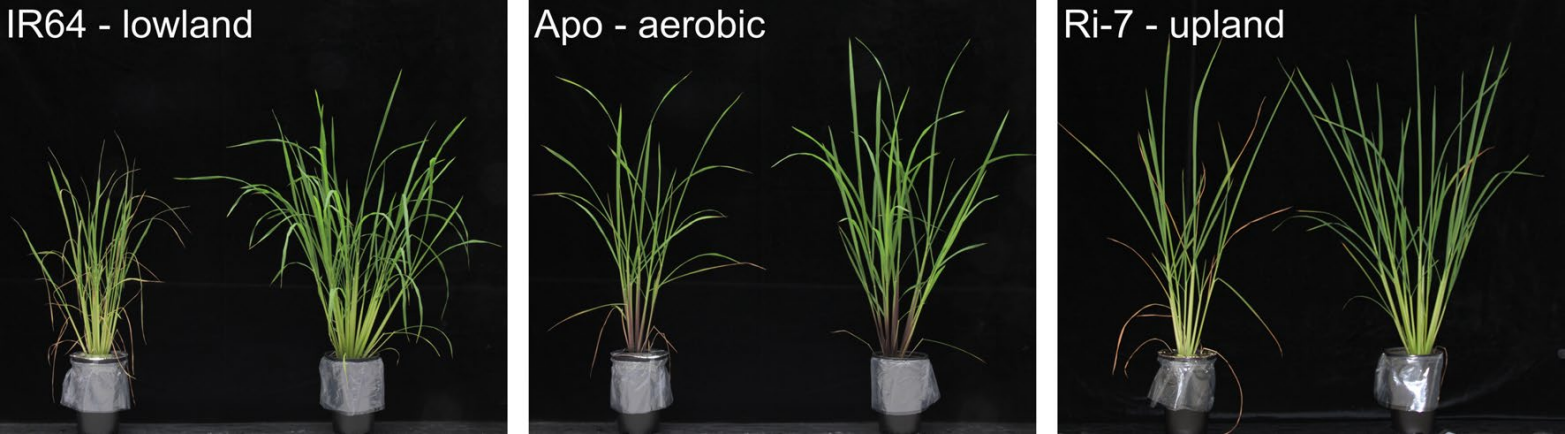
Effect of drought treatment on the shoot phenotype in the three rice varieties. Pictures of IR64 (left), Apo (middle) and Ri-7 (right) at the recovery time point (TP4, 2 days after re-watering) of sampling. In each picture, the stressed plant is on the left with its respective control on the right

At the end of the stress treatment, drought resulted in visible growth reduction, with clear differences between the varieties (Fig. 3). Overall, under control conditions, IR64 accumulated a higher leaf biomass than the other two varieties, at all TPs (Fig. 2d–f). Under drought, IR64 displayed a significant reduction in leaf biomass compared with the control, starting at TP1 (Fig. 2g). This reduction remained significant and stable at TP2, and sharply increased at TP3 and TP4, when IR64 reached the largest reduction in leaf biomass (− 77.6%) of the three varieties. Leaf biomass of Apo was significantly reduced from TP2 onwards (Fig. 2e), and the difference with the control increased more gradually than in IR64 at TP3 and TP4 (Fig. 2h). Ri-7 did not show a reduction in leaf biomass under drought in the first two TPs. In Ri-7, the decrease in leaf biomass started at TP3 (Fig. 2f) and this continued at TP4 (Fig. 2i). Under severe drought (TP3), strong leaf rolling was observed in all plants of all three varieties. Rolling fully recovered after re-watering (TP4), but with leaf tips showing necrotic areas (Fig. 3). These necrotic areas were discarded and not considered for leaf biomass determination at TP4. The lowest reduction in leaf biomass between TP3 and TP4 was displayed by Apo (− 12.7%), followed by Ri-7 (− 23.9%) and IR64 (− 32.7%).

All varieties showed a similar trend of biomass reduction for stem dry weight (DW), but with less significant differences than for leaf DW (Suppl. Table S1). Stressed plants of all varieties also had a significantly lower root DW compared with control plants, but only at TP4. Interestingly, in IR64, drought caused a significant reduction in tiller number under drought, starting at TP2, while this reduction started later in Apo and Ri-7 (Suppl. Table S1).

Taken together, these results highlight contrasting constitutive growth rates among the three varieties under control conditions as well as different growth responses to increasing drought and upon recovery.

### Overall effect of drought and re-watering on metabolism and oxidative stress status

To get insight into the drought-induced changes in leaf metabolism and oxidative stress status, we analyzed the levels of primary metabolites (29), nutrient ions (3) and oxidative stress markers/enzymes (7), at all time points. The levels (mean ± SD) of the 39 variables under control and drought treatments are shown in Suppl. Table S2 and S3, respectively.

First, we examined the effect of variety and plant development (TPs) on the metabolic, nutrient and oxidative stress variables under optimal conditions using principal component analysis (PCA) (Suppl. Fig. S3). The first two principal components (PCs) explained more than 50% of the total variation but did not clearly separate the samples by TPs or variety. Only TP1 and TP4 samples, with maximum plant age difference (8 days), did not overlap along PC1.

**Fig. 4:**
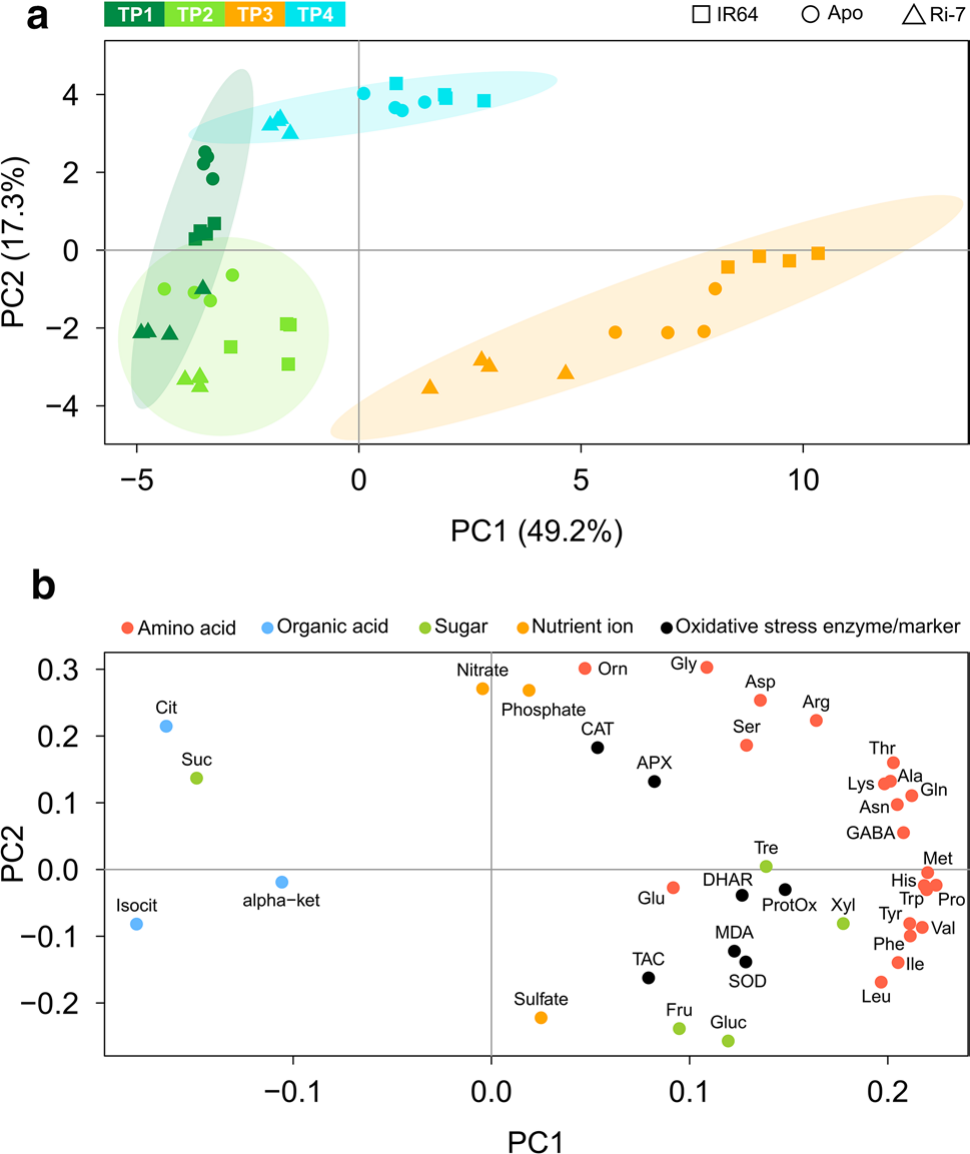
Principle component analysis of the effect of transient drought on metabolites, nutrient ions, and oxidative stress markers/enzymes in the three rice varieties. Principal component analysis score plot (**a**) based on the drought values of metabolites, nutrient ions and oxidative stress markers/enzymes of the biological replicates of IR64 (square), Apo (circle) and Ri-7 (triangle) at the four time points (TP1, TP2, TP3, TP4). Biological replicates are colored according to the time point of sampling (TP1: dark green; TP2: light green; TP3: orange; TP4: light blue). Ellipses represent the 95% confidence level for each TP. Loading plot (**b**) of the 39 variables colored based on their class (amino acid: red; organic acid: light blue; sugar: green; nutrient ion: orange; oxidative stress marker/enzyme: black)

For the PCA with the drought values of the same 39 metabolic, nutrient and oxidative stress variables, the first two PCs explained ~ 67% of the total variation, with PC1 alone explaining 49.2% (Fig. 4a). Along PC1, the samples followed a distribution from left to right in accordance with the increasing severity of drought from TP1 to TP3. This suggests that PC1 represents the leaf metabolic and oxidative stress signature of drought. On this PC, TP1 (early-mild stress) samples of the three genotypes did not clearly separate from the ones of TP2 (mild-severe stress). Samples of TP3 (severe stress) clearly separated from the others and displayed a wide distribution with Ri-7 replicates being closest to TP1 and TP2 samples (suggesting a smaller change in the metabolic and oxidative/antioxidative response profile), followed by replicates of Apo and IR64 (largest change in the profile), respectively. Samples of TP4 (stress recovery) are in between TP2 and TP3 along PC1, albeit a bit higher along PC2. Loadings of the variables on PC1 (Fig. 4b) showed that less severe drought (TP1 and TP2) is associated with elevated tricarboxylic acid (TCA) cycle intermediates (isocitrate, citrate, and α-ketoglutarate) and sucrose. In contrast, severe drought (TP3) is associated with elevated levels of all amino acids, oxidative stress markers/enzymes and of glucose, fructose, trehalose, and xylose. Interestingly, along PC2 (Fig. 4a), explaining 17.3% of the total variation, there is a more marked separation between drought-treated (TP2, TP3 and only partially TP1) and re-watered samples (TP4), suggesting that PC2 represents metabolic and oxidative stress differences between drought and recovery. Loadings of the variables on PC2 (Fig. 4b) showed that re-watered samples (TP4) displayed increased levels of specific amino acids (mainly Orn, Gly, Asp, Arg and Ser), the nutrient ions nitrate and phosphate, citrate and sucrose, and the antioxidant enzymes CAT and APX.

### Stress-induced responses in leaf metabolites and oxidative stress markers/enzymes

To analyze drought-induced changes in primary metabolism and oxidative stress status in the leaves of the three rice varieties during stress imposition and after re-watering, we conducted a two-way ANOVA at each TP using drought treatment (Treat) as one factor and variety (Var) as the second (exact *P* values for each factor are reported in Suppl. Table S4). Table 1 shows that the number of metabolites and oxidative stress markers/enzymes that significantly changed (Bonferroni-corrected *P* < 0.05) because of the drought treatment or the interaction (Int) between variety and treatment. This number gradually increased between TP1 (*n* = 15; Treat: 11; Int: 4) and TP2 (*n* = 23; Treat: 18; Int: 5) before drastically increasing at TP3 (*n* = 56; Treat: 35; Int: 21) and decreasing (*n* = 42; Treat: 26; Int: 16) again after re-watering (TP4). The number of metabolites and oxidative stress markers/enzymes that were significantly changed because of the effect of the variety remained relatively stable across the four TPs (*n* = 20, 23, 26, 24 from TP1 to TP4, respectively). These numbers show that at the earlier and less intense stress TPs (TP1 and TP2), the effect of varietal differences on the leaf metabolic and oxidative stress profile was similar to the effect of the drought. However, at TP3 and TP4, the effect of the drought treatment on the leaf metabolic and oxidative stress profiles exceeded that of the variety differences. These observations support the PCA results that show a more marked separation between varieties at TP3 and TP4 on PC1, representative of drought, than between TP1 and TP2 samples, which mostly overlapped on the same PC (Fig. 4a).

**Table 1:**
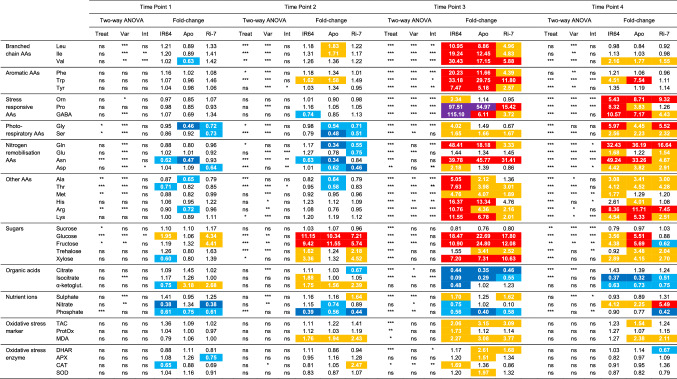
The effect of drought on the leaf levels of metabolites, nutrient ions and oxidative stress markers/enzymes of the three rice varieties at vegetative stage

Mean fold-change (drought over control) values of the 39 variables at each time point (Time Point 1–4) for the three varieties. Bonferroni-corrected *P* values (*** = *P* < 0.001; ** = *P* < 0.01; * = *P* < 0.05; ns = not significant) for the factors (*Var* variety, *Treat* treatment, *Int* interaction) of the two-way ANOVA analysis are reported for each time point. Fold-change (fold) values are highlighted in different colors: fold ≤ 0.5 (dark blue); 0.5 < fold ≤ 0.75 (light blue); 1.5 ≤ fold ≤ 5.0 (yellow); 5.0 < fold ≤ 50 (red); fold > 50 (purple)

To allow for a more detailed look at the drought-induced metabolic and oxidative stress changes in the leaves of the three rice varieties during stress and recovery, the mean fold-change values (drought over control) of each variable at each TP were calculated (Table 1). Particularly, we focused only on the variables showing high deviations from control (in Table 1, fold-change values are highlighted with colors only if < 0.75 or > 1.5).

At early-mild (TP1) and mild-severe (TP2) stress intensities, just few amino acids were affected by drought, with a marked decrease (~ 0.5-fold or lower) in amino acids associated with photorespiration (Gly and Ser) and nitrogen remobilisation (Gln and Asn) at TP2, especially in leaf samples of Apo and, to a lesser extent, of Ri-7. Different from the two earlier TPs, almost all the amino acids strongly increased upon severe drought (TP3) with the highest fold-change values detected in IR64, followed by Apo and Ri-7. In particular, the stress-responsive amino acid Pro increased massively (almost 100-fold) in IR64, but less in Apo (55-fold) and Ri-7 (15-fold). In IR64, GABA showed the highest increase among all metabolites (115-fold); whereas, it increased tenfold less in Apo and Ri-7. After re-watering (TP4), the majority of the amino acids still showed increased values from control, but to a lower extent and with less distinct differences between varieties than at TP3. Nevertheless, it is interesting to note that, at TP4, the stress-responsive amino acid Pro was still significantly higher in the stressed leaf samples of IR64, but not in the leaves of the other two varieties.

The drought response of organic acids followed the opposite trend of the amino acids. Except for a moderate (~ two- to ~ three-fold) increase of α-ketoglutarate at TP1 (in Apo) and TP2 (in IR64), no significant changes in organic acids were observed until severe stress (TP3) when the levels of the TCA cycle intermediates, citrate and isocitrate, strongly decreased (fold < 0.5) in the leaves of all varieties. A similar strong decrease for isocitrate was observed also after re-watering (TP4) in all three varieties.

Different from the previous two classes of metabolites, sugars displayed a more gradual increase from early-mild (TP1) to severe (TP3) stress. At TP1, this increase was already quite marked (~ four-fold) in leaves of Ri-7 for fructose and glucose with the latter showing an increase in IR64 too, albeit less strong (~ two-fold). At TP2, the three varieties displayed a high and quite similar increase in glucose (7- to 11-fold) and fructose (6- to 12-fold) compared with the control. These values increased even more at TP3 before decreasing again after re-watering (TP4) when the two sugars still showed a higher value in the stressed (and re-watered) leaves of IR64 and Apo, but not of Ri-7. Sucrose did not show any important change from control at the four TPs; whereas, the hemicellulose-derived sugar xylose displayed a similar increase as the other sugars in all varieties at severe drought (TP3) (7- to 10-fold) which decreased at TP4 (two- to four-fold).

Among the nutrient ions, phosphate decreased almost equally in the leaf samples of the three varieties from TP1 to TP3; whereas, at TP4, it showed a strong decrease only in the leaves of Ri-7.

In the oxidative stress markers and enzymes, no significant changes were observed on the first two TPs except from a slightly significant increase of CAT in Ri-7 at mild-severe stress (TP2). At TP3, the oxidative stress markers and enzymes showed a significant and strong upregulation by drought. ProtOx more markedly increased in IR64 leaves. The levels of the lipid peroxidation product malondialdehyde (MDA) and of total antioxidant capacity (TAC) similarly increased (two- to three-fold) in the leaves of the three varieties. Among the antioxidant enzymes, DHAR showed a higher activity in the leaves of Apo (> 2.5-fold) and Ri-7 (> 1.5-fold) but not of IR64. Apo leaves also displayed an increase (~ two-fold) in the activity of SOD, APX (~ 1.5-fold), while CAT activity increased (> 1.5-fold) in IR64 leaves. After re-watering (TP4), oxidative stress markers and enzymes were not significantly different from the control, except for a slightly lower activity of DHAR in the leaves of Ri-7.

Overall, these results show that the different classes of primary metabolites (amino acids, sugars, organic acids), nutrient ions and oxidative stress markers/enzymes showed a similar response to increasing drought and re-watering in the leaves of the three varieties. However, the intensity of this response was markedly different between the varieties and, considering each TP separately, we identified specific varietal responses for certain metabolites and oxidative stress markers/enzymes.

### Metabolic signatures of leaf senescence

**Fig. 5:**
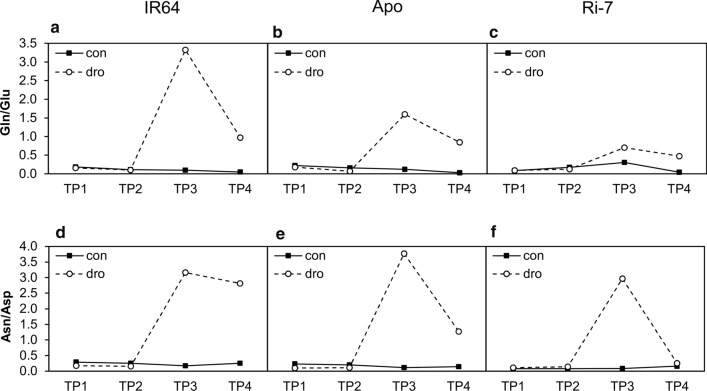
Drought-induced variation of the leaf senescence markers Gln/Glu and Asn/Asp ratios in the three rice varieties. Mean values of the glutamine to glutamate (Gln/Glu) and asparagine to aspartate (Asn/Asp) ratios at the four time points (TP1, TP2, TP3, TP4) under control (con) and drought (dro) conditions in IR64 (**a**, **d**), Apo (**b**, **e**) and Ri-7 (**c**, **f**)

Gln and Asn are two amino acids involved in nitrogen recycling and export from senescent leaves (Chrobok et al. 2016). To evaluate the level of leaf senescence among the varieties, we calculated the ratio of glutamine to glutamate (Gln/Glu) and asparagine to aspartate (Asn/Asp) (Watanabe et al. 2013) for each variety at the four TPs (Fig. 5). High Gln/Glu and Asn/Asp ratios represent a signature of nitrogen recycling activity. Under control conditions, the Gln/Glu and Asn/Asp ratios were consistently low (ratio < 0.3) for all varieties at all TPs. Both ratios did not respond to the drought treatment at early-mild (TP1) and mild-severe (TP2) stress intensity. At severe stress (TP3), however, the Gln/Glu ratio strongly increased in leaves of IR64 and to a lesser extent in leaves of Apo and Ri-7. The Asn/Asp ratio showed a similar marked increase. After re-watering (TP4), both ratios decreased again with leaves of Ri-7 showing the lowest values (almost the same as for control for Asn/Asp and slightly higher for Gln/Glu), followed by leaves of Apo and of IR64 that still had a higher Asn/Asp ratio than in the control.

The changes in the Gln/Glu and Asn/Asp ratios suggest that premature drought-induced leaf senescence only occurred-in all varieties but to a higher extent in IR64-under severe drought (TP3) and decreased again upon re-watering.

### Qualitative comparison of leaf metabolic and oxidative stress profiles between the vegetative and reproductive stage

The three rice varieties had also been included in a large field trial conducted at the International Rice Research Institute (IRRI), Philippines, during the 2013 dry season (Kadam et al. 2018). In the field trial, drought exposure in the reproductive stage induced a decrease in grain yield of 27.1% in IR64 and 20.3% in Ri-7 whereas Apo did not exhibit grain yield reduction but a slight increase of 5.8%.

We used fold-change analysis to qualitatively compare the drought-induced differences in the leaf metabolic and oxidative stress profile of the three rice varieties in the field, during the reproductive stage, and in the growth chamber, during the vegetative stage. For the field experiment, we only had a single replicate for 33 metabolic and oxidative stress variables (Suppl. Table S5), but each sample was composed of the flag leaves of 24 different plants (as described in the Materials and methods). Table 2 shows the drought-induced fold-changes of the 33 primary metabolites and oxidative stress markers/enzymes detected in the flag leaves of the three varieties at the reproductive stage. As in Table 1, we focused only on variables showing high deviations from control (fold-change decrease < 0.75 or increase > 1.5). Also in the field experiment, almost all amino acids showed a drought-induced increase (between 1.5- and 5-fold) in the flag leaves of IR64 (10 amino acids) and Ri-7 (9 amino acids) but not of Apo (2 amino acids). This increase is less marked than at TP3 in the vegetative stage but opposite to the decrease displayed by the three varieties at early-mild (TP1) and mild-severe (TP2) stress during vegetative development (Tables 1 and 2). The decrease in organic acids observed under severe drought (TP3) in the vegetative stage, also occurred in the field in the reproductive stage although less strong for IR64 and Apo. Surprisingly, sugars did not show any important deviation from control in the reproductive stage (Table 2). This is in contrast with what we observed in all genotypes at mild-severe (TP2) and severe (TP3) drought during vegetative development (Table 1). Interestingly, oxidative stress markers and enzymes (with the exception of total antioxidant capacity, TAC) showed an overall strong upregulation in all varieties in the reproductive stage, even stronger than observed under severe drought (TP3) in the vegetative stage (Tables 1 and 2). Malondialdehyde (MDA) increased in IR64 and Ri-7 (> two-fold) but not in Apo while ProtOx increased in Ri-7 and Apo (~ two-fold). Apo displayed a very strong increase in all the antioxidant enzymes (between 2.5- and 7-fold), and it was the only genotype showing a marked increase in DHAR (~ 2.5-fold) and APX (~ 7-fold). CAT and SOD increased strongly in all three varieties with Apo displaying the highest values for the first enzyme and Ri-7 for the second (Table 2).

**Table 2:**
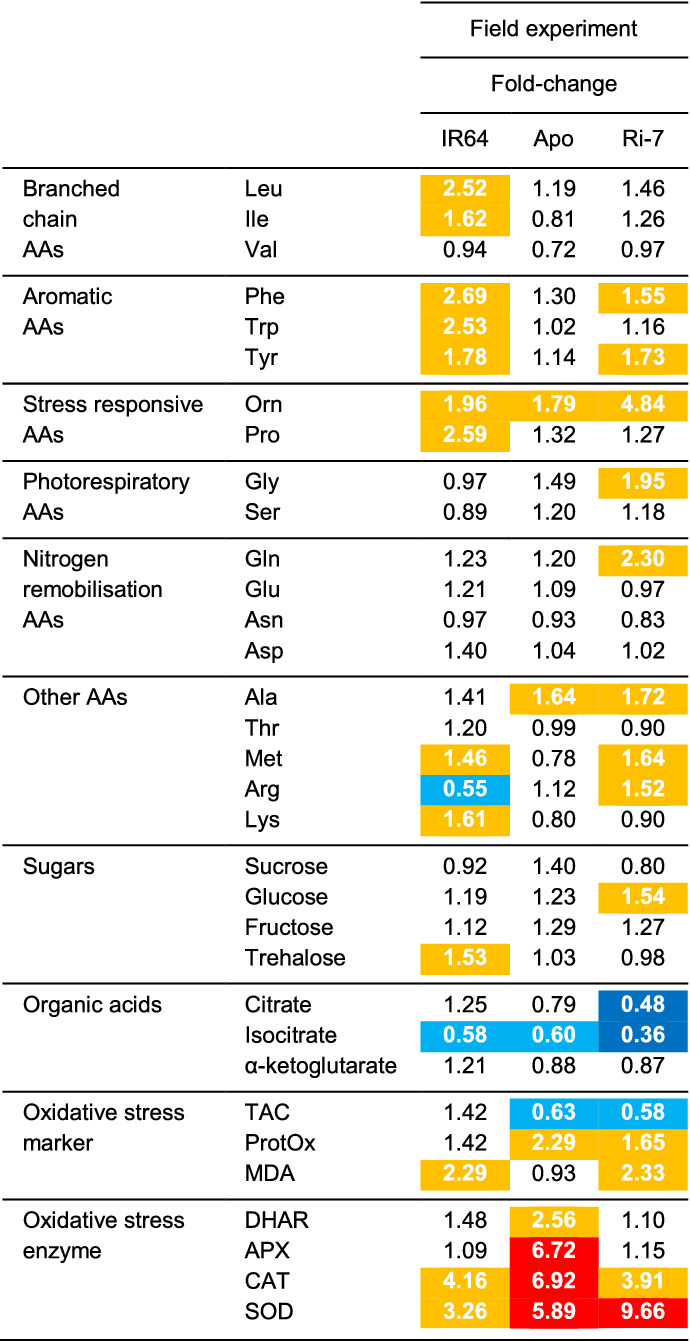
The effect of drought on the flag leaf levels of metabolites and oxidative stress markers/enzymes of the three rice varieties at the reproductive stage

Fold-change (drought over control) values of 33 variables for the three varieties at the reproductive stage. Fold-change (fold) values are highlighted in different colors: fold ≤ 0.5 (dark blue); 0.5 < fold ≤ 0.75 (light blue); 1.5 ≤ fold ≤ 5.0 (yellow); fold > 5 (red)

In summary, the metabolic and oxidative stress profiles of the three varieties in the reproductive stage showed a similar, but less marked, response to drought in amino acids (increase) and organic acids (decrease) as when exposed to severe drought (TP3) during vegetative development. This response was different in the sugars (no change) and in the activity of antioxidant enzymes (strong increase).

## Discussion

### Drought in the vegetative stage has a stronger impact on the ‘water-spender’ IR64 than on the ‘water-savers’ Apo and Ri-7

The transpiration differences between the three varieties suggest that the high and sustained water consumption of IR64 under water-limiting conditions exposed the lowland variety to a more prolonged and severe drought than the other two varieties, which displayed a more limited water use (Fig. 1). These results are in agreement with a study by Ouyang et al. (2017) who showed that IR64 has higher stomatal conductance and lower transpiration efficiency than Apo and Ri-7, both under well-watered and mild drought conditions. Our findings also point towards the constitutive higher leaf biomass in IR64, under optimal growth conditions (Fig. 2d–f and Suppl. Table S1), as an additional cause for its higher water loss through transpiration, especially during the first days of water withholding (until TP1) (Fig. 1). This may be further aggravated by the fact that the pot experiment probably restricted the investment in below ground biomass under drought as indicated by the non-significant differences in root biomass between control and stressed plants of the three varieties at TP1-TP3 (Suppl. Table S1).

The higher water consumption of IR64 compared to Apo and Ri-7, which was also maintained after water withholding started, was therefore likely the cause of the extremely strong dehydration (to about 25% RWC) suffered by the lowland variety after 10 days (TP3) of stress (Fig. 2a). Keeping a high cellular hydration under drought is crucial to maintain essential metabolic functions and to adapt to the prevailing stress conditions (Obata and Fernie 2012; Obidiegwu et al. 2015). The lower RWC in IR64 than in Apo and Ri-7 (~ 70% RWC) at TP3 (Fig. 2b, c) might have increased the incidence of leaf cell death (leaf necrotic lesions were visible in all genotypes at this TP) more in the lowland variety than in the aerobic and upland varieties. This hypothesis is supported by the stronger leaf biomass loss observed in IR64 than in Apo and Ri-7 after re-watering (TP4) (Figs. 2d–i and 3) and, also, by the levels of the nitrogen remobilization markers, Gln/Glu and Asn/Asp (Fig. 5), discussed below.

### Relationships between growth dynamics, primary metabolism, and oxidative stress status

Leaf growth reduction in response to drought is one of the mechanisms used by plants to limit the expansion of their evaporation surface and it generally occurs before reduction of photosynthesis (Hummel et al. 2010; Claeys and Inze 2013). Different rates of leaf expansion under drought were observed before in rice genotypes with contrasting drought tolerance (Cabuslay et al. 2002; Parent et al. 2010) and confirmed by our leaf growth results (Fig. 2g–i). In our study, high transpiration values (Fig. 1) together with the absence of increased levels for the photorespiratory amino acids Gly and Ser (Table 1) in all the three varieties at both TP1 and TP2 support the presence of an active photosynthetic metabolism until severe stress (TP3). Indeed, drought-induced stomata closure should have strongly reduced photosynthesis and increased the activity of the photorespiratory pathway, resulting in higher production of Gly and Ser (Bauwe et al. 2010; Maurino and Peterhansel 2010; Hodges et al. 2016), that was observed only at TP3 (Table 1).

Maintaining photosynthetic active metabolism in combination with reduced growth often results in an increase in the concentration of carbohydrates in the leaves (Muller et al. 2011; Blum 2017). Reduced leaf growth occurred in IR64 at the earliest stage of stress (TP1) and only at more severe drought intensities in Apo (TP2) and Ri-7 (TP3) (Fig. 2g–i). Surprisingly, the early reduction of leaf biomass in IR64 was not associated with an increase in the concentration of the main sugars (Table 1). A possible explanation for this might be that, at early-mild drought (TP1), the source leaves of IR64 were still exporting carbon to the growing sink organs (young leaves, stems and roots) to sustain the higher biomass growth of this variety (Fig. 2d–f and Suppl. Table S1). Similarly, the low level of stress experienced by Apo at TP1 did not reduce its leaf growth (Fig. 2e and h) making Apo leaves net exporters of sugars that, indeed, did not show increased levels (Table 1). Like Apo, Ri-7 did not show a reduction in leaf biomass at TP1 (Fig. 2f and 2i) but its fructose and glucose levels markedly increased (Table1). Sugar-mediated osmotic adjustment, including accumulation of hexose sugars in mature leaves of rice (Luquet et al. 2008), is a response to drought that helps to sustain cell turgor, stomatal opening and photosynthesis, but at the expense of overall plant growth (Blum 2017). In Ri-7, the accumulation of fructose and glucose in mature leaves at early-mild drought (TP1) might represent a strategy of acclimation to drought that is associated with the lower biomass growth of this upland variety. This sugar-mediated early acclimation to drought in Ri-7 is possibly responsible for the tolerance of this variety.

Progression of drought severity from early-mild (TP1) to mild-severe (TP2) resulted in a sudden and strong increase of fructose and glucose (~ ten-fold) in Apo and IR64 and less marked (~ six-fold) in Ri-7 (Table 1). Despite the similar strong increase in sugars in the three varieties, they displayed a very different leaf growth response to drought at TP2. Fructose and glucose accumulation in Apo was associated with a marked reduction in leaf growth (Fig. 2h) and, therefore, it might represent an osmotic adjustment response to drought (Lilley and Ludlow 1996; Luquet et al. 2008; Blum 2017). At TP2, IR64 did not display a further increase in leaf biomass loss from TP1 (Fig. 2g), even though the higher severity of the stress induced a strong accumulation of carbohydrates in its mature leaves (Table 1). A possible explanation is that IR64 mature leaves stopped to act as carbon exporters to other sink organs. Confirming this hypothesis, IR64 was the only variety displaying a reduction of stem weight at TP2 (Suppl. Table S1). In rice, the excess photo-assimilates produced in leaves is stored in the stem as carbohydrate reserves that can be used to buffer leaf performance under stress, mainly at the reproductive stage (Yang et al. 2001; Morita and Nakano 2011; Wang et al. 2017). Our results suggest that, in IR64, non-structural carbohydrates stored in the stem might have been remobilized (starting from TP2) to stabilize leaf growth and provide osmotic protection under drought already at the vegetative stage. Nevertheless, the earlier use of these stem reserves, compared with the other two varieties (stem weight reduction in Apo and Ri-7 started only at TP3 and TP4, respectively), might have undermined stem vigor of IR64 and contributed to the early reduction in tiller number, starting at TP2 only in this variety (Suppl. Table S1). Different from IR64 and Apo, the increase of fructose and glucose in Ri-7 at TP2 was not coupled with any reduction in the biomass of leaves, stems or roots (Fig. 2i and Suppl. Table S1). This observation further supports the hypothesis of a sugar-mediated osmotic adjustment to drought as the origin for the high levels of the two sugars (already observed at TP1) in the leaves of Ri-7.

Prolonged and severe drought (10 days, TP3) caused a marked reduction in growth in all varieties (Fig. 2g–i and Suppl. Table S1). This reduction was reflected in a state of strong leaf metabolic alteration (Table 1). At this TP, the extremely high levels of almost all amino acids displayed by the three varieties were reported before to occur in leaves of many crop species exposed to drought (Krasensky and Jonak 2012; Obata and Fernie 2012), including rice (Todaka et al. 2017; Casartelli et al. 2018; Gayen et al. 2019; Melandri et al. 2020). This strong amino acid increase is associated with protein catabolism occurring during premature stress-induced leaf senescence (Araújo et al. 2011; Watanabe et al. 2013; Hildebrandt et al. 2015). The hypothesis of a strong catabolic activity at severe drought (TP3) is supported by the increased asparagine to aspartate (Asn/Asp) and glutamine to glutamate (Gln/Glu) ratios observed in the three varieties at this sampling point (Fig. 5). In senescent leaves, protein degradation-derived aspartate and glutamate are converted to asparagine and glutamine to act as transport molecules for long-distance nitrogen remobilisation through the phloem (Watanabe et al. 2013; Avila-Ospina et al. 2014). A further confirmation of a high catabolic activity at severe drought (TP3) in the three varieties was represented by the shared decrease in the level of two TCA cycle intermediates, isocitric and citric acid (Table 1). The TCA cycle is involved in the production of metabolic intermediates used in biosynthesis elsewhere in the cell (Araújo et al. 2012). The reduction of organic acids of the TCA cycle was observed before in rice seedlings and leaves exposed to drought (Todaka et al. 2017; Casartelli et al. 2018; Melandri et al. 2020) and considered as an indicator of reduced biosynthesis under stress. Similar to TP2, at severe drought (TP3), the three varieties displayed a comparable and very high accumulation of fructose and glucose (Table 1), but, differently from the previous TP, this sugar increase is likely the result of the initiation of starch degradation originating from drought-induced senescence (Wingler et al. 2006; Pinheiro and Chaves 2011). Despite the similar and shared state of metabolic changes observed among the three varieties at TP3, the extent of this change was more severe in IR64, followed by Apo and Ri-7 as overall indicated by PCA (samples of TP3 in Fig. 4a). The different level of stress severity for the three varieties at TP3 is particularly evident considering the marker of leaf senescence Gln/Glu (Fig. 5a–c). In addition, the different levels of certain metabolites, such as Pro and GABA, are also indicative of the different stress status of the three varieties at TP3. Accumulation of Pro under drought has often been reported in the literature (Krasensky and Jonak 2012; Fàbregas and Fernie 2019), also in rice (Raorane et al. 2015; Todaka et al. 2017; Melandri et al. 2020), and it was proposed as a possible response aimed to counteract the high ROS-induced oxidative stress caused by dehydration (Verslues and Juenger 2011; Nakabayashi and Saito 2015). GABA strongly accumulates, like Pro, during many abiotic stresses (Obata and Fernie 2012; Fàbregas and Fernie 2019). Even if its role under stress remains unclear, GABA has been proposed as a regulator of osmolarity and coordinator of the carbon–nitrogen balance under conditions of carbon limitation (Hildebrandt et al. 2015; Michaeli and Fromm 2015; Fàbregas and Fernie 2019). In this study, at severe drought (TP3), the extremely high levels of Pro and GABA in the leaves of IR64 (~ 100-fold), followed by Apo and Ri-7 (Table1) seem to represent markers for the different levels of leaf dehydration and senescence of the three varieties at this TP (Figs. 2a–c, 5a–c).

Differences in the level of oxidation and antioxidant activity between the three varieties were only observed at TP3 under severe drought (Table 1). It has been shown that under persistent water limitation, drought-induced stomatal closure increases ROS production as a result of excess light and reduced CO_2_ assimilation (Asada 2006; Miller et al. 2010). Among the three varieties, Apo displayed simultaneously the highest levels of DHAR, SOD, APX and total antioxidant capacity (TAC) suggesting a broader antioxidant capacity under drought than the other two varieties. DHAR regenerates oxidized ascorbate with reduced glutathione as electron donor (Das and Roychoudhury 2014) through the ascorbate–glutathione pathway, the main redox hub in plants (Foyer and Noctor 2011; Foyer 2018). Increased activity of DHAR and SOD under drought was described before in rice seedlings of different cultivars (Selote and Khanna-Chopra 2004; Sharma and Dubey 2005). SOD converts highly oxidative superoxide (O_2_^−^) to less harmful H_2_O_2_ (Halliwell 2006). A marked increase in the activity of SOD, observed only in Apo, could represent a safeguarding mechanism to generate H_2_O_2_ that, in turn, could trigger a wider array of antioxidant defences.

After re-watering, the differences in the leaf metabolic and oxidative stress profiles were more similar to the control than to the severe drought treatment (TP3) and with less marked differences between varieties (Fig. 4a; Table 1). This shows that a similar recovery from stress occurred in the three varieties and suggests that their leaf biomass reduction (− 77.6% in IR64, − 58.4% in Ri-7 and − 50.2% in Apo) after re-watering is entirely due to the differences in the metabolic and antioxidative responses during drought.

### During reproduction specific metabolic and antioxidative responses to drought may be less effective

The qualitative comparison between leaf metabolic and oxidative stress profiles of the three varieties in the two different phenological stages (Tables 1 and 2) suggests that plants in the field experienced a stress intensity lower than severe drought (TP3) under controlled conditions. The flag leaf metabolic profiles of the three varieties in the reproductive stage (in the field; Table 2) had a similar signature of drought-induced leaf senescence (increased amino acids, but less than at TP3) and of lower biosynthetic activity (decreased organic acids of the TCA cycle like at TP2) as during the vegetative stage. In the reproductive stage, this signature was particularly marked in IR64 and Ri-7 but much less so in Apo. Unlike at TP2 and TP3 in the vegetative stage, in the reproductive stage the levels of sugars did not increase compared with the control (Tables 1 and 2). This might be caused by a stable sugar export under drought during flowering/grain filling when the flag leaf is the most important source of assimilates for the developing panicles and, thus, for yield stability (Yoshida 1972; Biswal and Kohli 2013). Exporting sugars (osmotic adjustments not possible) in a context of high drought-induced stress (lower photosynthetic capacity), might have exposed the flag leaves of the three varieties to enhanced generation of ROS and oxidative damage (Suzuki et al. 2012; Noctor et al. 2014). This hypothesis is supported by the high levels of the lipid peroxidation product malondialdehyde (MDA) in IR64 and Ri-7, and by high ProtOx in Apo and Ri-7 (Table 2). All three varieties displayed a marked increase in the activity of antioxidant enzymes, with higher values than at severe drought (TP3) during the vegetative stage (Tables 1 and 2). Among the three varieties, Apo displayed the strongest and most inclusive (DHAR, APX, CAT and SOD) antioxidant response under drought also in the reproductive stage. This response might have helped Apo to minimize grain yield loss, unlike IR64 and Ri-7 in which less antioxidant enzymes (CAT and SOD) increased in activity. This hypothesis suggests that a strong antioxidant response to drought at the reproductive stage might be key for minimizing grain yield loss.

## Conclusions

The aim of the present research was to determine how drought-induced leaf metabolic and oxidative stress responses in drought-tolerant rice varieties differ from a susceptible genotype to better understand the mechanisms of adaptation to drought during the vegetative stage. The results show that a reduced use of water coupled with an early sugar-mediated osmotic acclimation in Ri-7 (upland rice) or a strong antioxidative response in Apo (aerobic rice) were equally effective in limiting drought-induced biomass losses in these two varieties compared with the susceptible variety IR64 (lowland rice). In the reproductive stage, only the variety with the highest antioxidative response, Apo, maintained a stable grain yield under reproductive phase drought in the field, suggesting that a strong antioxidant activity represents a crucial mechanism of tolerance to drought at this specific stage.

The improved understanding of the physiological, metabolic and antioxidative response strategies associated with drought tolerance in rice at the vegetative stage generated by this study, provides a framework for the exploration of the genetic control of these mechanisms and their exploitation in breeding for new varieties adapted to water-limited environments.

### Author contribution statement

GM, CR and HB conceptualized and managed the project. GM, HAE, KF and DJ conducted the metabolite and oxidative stress analyses. GM analyzed the data. GM and HB wrote the paper with inputs from CR, GB, and HA. All authors read and approved the final manuscript.

## Supplementary Information

Below is the link to the electronic supplementary material.

Supplementary file1 (PDF 653 KB)

Supplementary file2 (PDF 452 KB)

Supplementary file3 (PDF 3733 KB)

## Data Availability

The datasets generated for this study are included as electronic supplementary material. Additional data will be made available on reasonable request.

## Abbreviations

APX: Ascorbate peroxidase
CAT: Catalase
DHAR: Dehydroascorbate reductase
PCA: Principal component analysis
SOD: Superoxide dismutase
TCA: Tricarboxylic acid
TP: Time point
