# Supplementary material for: Drought tolerance in selected aerobic and upland rice varieties is driven by different metabolic and antioxidative responses
Source: Planta. 2021 Jun 25;254(1):13. doi: 10.1007/s00425-021-03659-4 (PMC8233253; doi:10.1007/s00425-021-03659-4)
Supplement: Supplementary file 1 — Supplementary file1 (PDF 653 KB) [file 425_2021_3659_MOESM1_ESM.pdf]

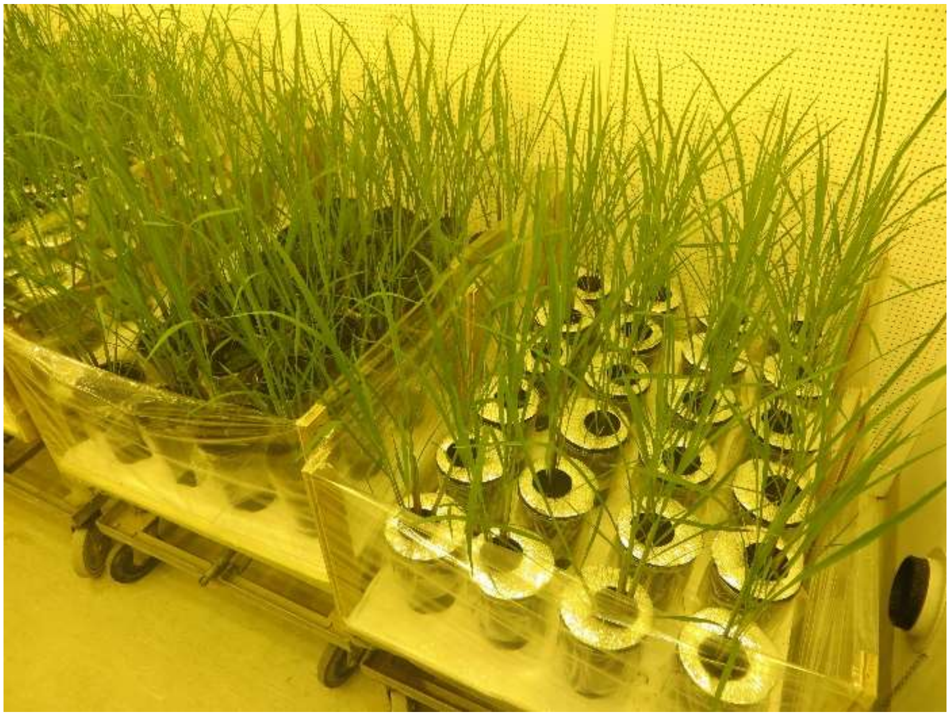

Fig. S1. The experimental setup.
Picture of two trolleys carrying the pots. Each trolley comprised 24 plants of the same variety under the same treatment. The trolley on the right contains plants that were exposed to drought. For these plants, the pots are covered with silver discs aiming to reduce the water evaporation under drought stress. The same discs are not present on the pots of the control plants on the other trolley (left).

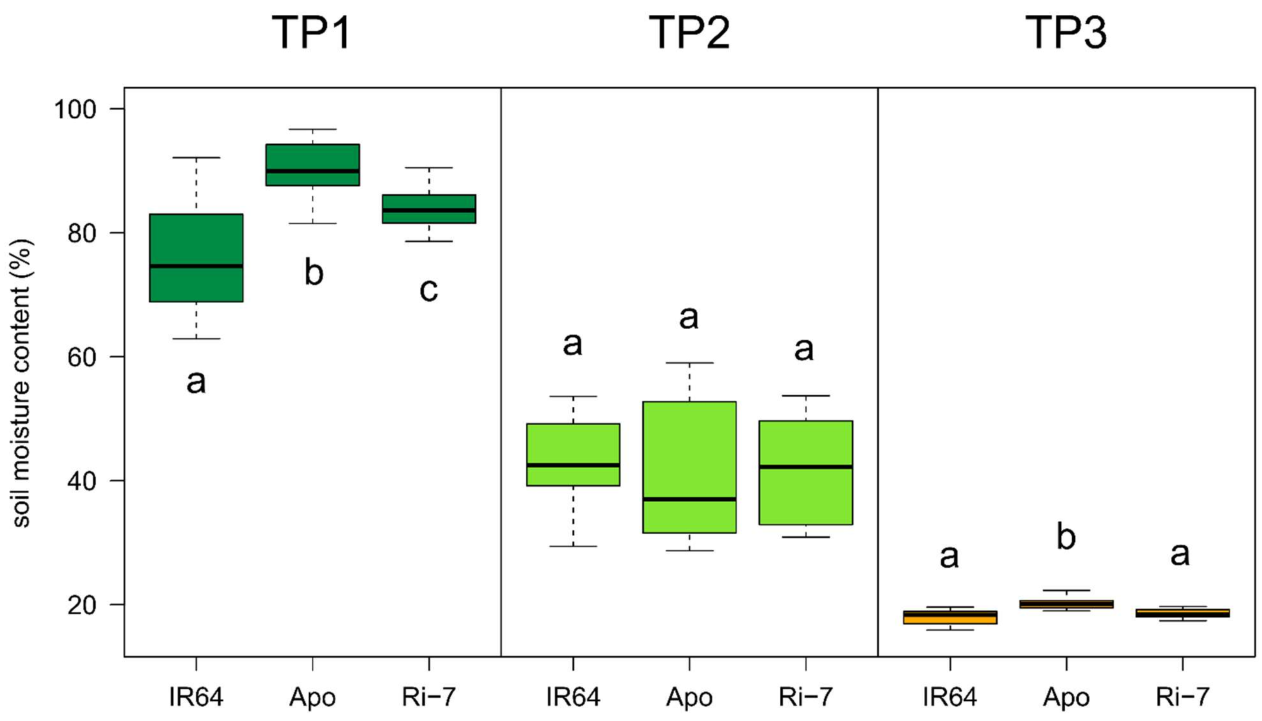

Fig. S2. Effect of the treatment on soil water content.
Boxplots representing variation of soil moisture content for IR64, Apo and Ri-7 at time points TP1, TP2 and TP3. Letters (a, b, c) indicate significant differences according to Tukey's test.

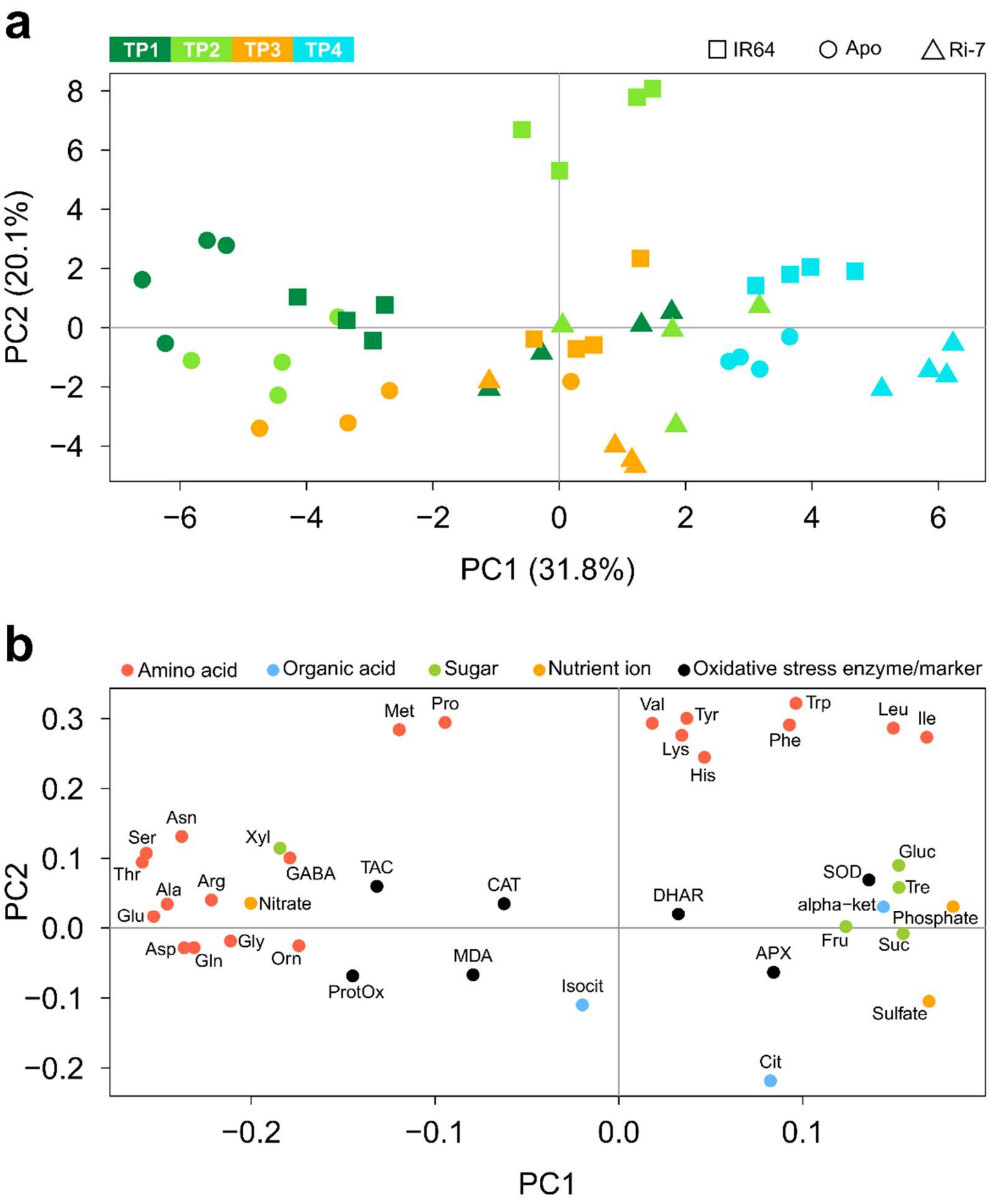

Fig. S3. Principal component analysis of leaf metabolites, nutrient ions and oxidative stress markers/enzymes in the control samples.

Principal component analysis score plot (a) based on the metabolite values of the biological replicates of IR64 (square), Apo (circle) and Ri-7 (triangle) at the four time points (TP1, TP2, TP3, TP4) under control conditions. Biological replicates are colored according to the time point of sampling (TP1: dark green; TP2: light green; TP3: orange; TP4: light blue). Loading plot (b) of the 39 variables colored based on their class (amino acid: red; organic acid: light blue; sugar: green; nutrient ion: orange; oxidative stress enzyme/marker: black).
