# Supplementary material for: Drought tolerance in selected aerobic and upland rice varieties is driven by different metabolic and antioxidative responses
Source: Planta. 2021 Jun 25;254(1):13. doi: 10.1007/s00425-021-03659-4 (PMC8233253; doi:10.1007/s00425-021-03659-4)
Supplement: Supplementary file 2 — Supplementary file2 (PDF 452 KB) [file 425_2021_3659_MOESM2_ESM.pdf]

Sugar quantifications by HPLC

Sugar (sucrose, fructose, glucose, trehalose and xylose) levels were measured as described by Bentsink *et al.* (2000), with minor modifications. Approximately 10 mg (DW) of freeze-dried material were homogenized in 1 mL of methanol (80% v/v) and 400 mg L^{-1} melezitose (internal standard). Samples were incubated in a water bath (76°C) for 15 min and the extract was dried using a Speedvac (room temperature, Savant SPD121P). The residue was resuspended in 1 mL MQ water and thoroughly vortexed and centrifuged for 5 min at 17000 g in an Eppendorf centrifuge. The supernatant was diluted (10x) in MQ water and injected into a Dionex HPLC system (ICS 5000 $^{+}$ DC), using a CarboPac PA 1, 4 x 250 mm column preceded by a guard column (CarboPac PA 1, 4 × 50 mm). Mono-, and disaccharides were separated by was a linear gradient from 25 to 100 mM NaOH for 15 minutes followed by a washing step of 5 minutes with 500 mM NaOH and an equilibration step of 15 minutes with 20 mM NaOH. Flow rates were 1 mL min^{-1} throughout the run. Peaks were identified by co-elution of external standards. The final quantity of the sugars was corrected by means of the internal standard and concentration was expressed in $mg \cdot L^{-1} \cdot g^{-1}$ of DW.

Nutrient anions and organic acids measurements by HPLC

The levels of nutrient anions (phosphate, nitrate and sulfate) and organic acids (citrate, isocitrate and α-ketoglutarate) were determined as described by He *et al.* (2014), with minor modifications. Approximately 5 mg (DW) of freeze-dried material was boiled at 100°C for 15 min in 1.0 mL 0.5 M HCl and 50 mg L-1 trans-aconitate (internal standard). After centrifuging for 2 min at 17000 g, 200 µL of the supernatant was transferred to an HPLC-vial. Samples were injected into a Dionex ICS2500 system with an AS11-HC column and an AG11-HC guard column and eluted with NaOH. The elution procedure was a linear gradient from 25 to 100 mM NaOH for 15 minutes followed by a washing step of 5 minutes with 500 mM NaOH and an equilibration step of 15 minutes with 5 mM NaOH. Flow rates were 1 mL min-1 throughout the run. Contaminating anions in the eluents were removed using an ion trap column (ATC), installed between the pump and the sample injection valve. Nutrient anions and organic acids were determined by conductivity detection. Background conductivity was decreased using an ASRS suppressor, with water as counterflow. Peaks were identified by co-elution of external standards. Final quantities were corrected by means of the internal standard and concentration were expressed in $mg \cdot L^{-1} \cdot g^{-1}$ of DW.

Amino acid detection and quantification using UPLC-MS/MS

Targeted analysis of individual amino acids was performed according to Carreno-Quintero *et al.* (2014) with modifications. 500 µL of extraction buffer (80% ethanol and isotopically labelled internal standard d3-Ala at a concentration of 20 pmol/µL) were added to 7 mg (DW) of leaf powdered freeze-dried material. The mixture was vortexed and sonicated for 10 min. After 5 min of centrifugation at 21,000 rcf the supernatant was transferred into a new tube. The extraction was repeated using the same volume of extraction buffer but without internal standard and the two supernatants were combined. The extract was then filtered using a Minisart SRP4 0.45 µm filter

(Sartorious-Stedim biotech, France). 10 μL of the sample was derivatized with 20 μL of 6-aminoquinolyl-N-hydroxysuccinidyl carbamate in 70 μL borate buffer using the AccQ•TagTM Ultra Derivatization kit according to the instructions of the manufacturer (Waters). The solution was vortexed for several seconds. After incubation at room temperature for 1 min the sample was heated at 55°C for 10 min. The derivatized amino acids were separated on the Waters Acquity UPLC system equipped with a binary solvent manager, an autosampler, a column heater, and interfaced to tandem quadrupole detector. The separation column was a Waters AccQ•TagTM Ultra (2.1 mm i.d. x 100 mm, 1.7 μm particles). Two mobile phases were used: AccQ•TagTM Ultra Eluent A1 (A) and AccQ• TagTM Ultra Eluent B (B). The flow rate was set at 0.7 mL/min and the separation gradient was set as follows: isocratic elution at 0.1% B for 0.54 min; 5.2 min linear gradient to 9.1% B, 2 min linear gradient to 21.2% B and 7.74-8.04 min to 59.6% B; 8.05-8.64 min isocratic column wash at 90.0% B and column equilibration for initial conditions for 0.83 min. The eluate was introduced into electrospray ion source (ESI) of triple quadrupole Xevo TQ mass spectrometer (Waters) operating at following conditions: source/desolvation temperature (150/600˚C), capillary voltage 1.5kV, cone voltage 30V, cone/desolvation gas flow (50/1000 L/hr), collision gas flow (0.15ml/min) and collision energy 20eV. The quantification of 21 derivatized amino acids (Ala, Ser, Pro, Val, Thr, Ile, Leu, Asp, Glu, Met, His, Phe, Arg, Tyr, Lys, Gly, GABA, Asn, Gln, Trp and Orn) was performed in multiple reaction monitoring (MRM) by isotope dilution method, using standard [$^2H_3$]-Ala in calibration curve and real samples. The software MassLynx^{TM} (Waters) was used to operate the instrument, acquire and process the MS data. The concentration levels were expressed in pmol·μL^{-1}·g^{-1} of DW.

Quantification of malondialdehyde, protein oxidation and total non-enzymatic antioxidant capacity

Malondialdehyde (MDA) content was assayed according to Hodges *et al.* (1999). For each sample 50 mg of leaves (FW) were homogenized in 80% (v/v) ethanol using a MagNA Lyser (Roche, Vilvoorde, Belgium). After centrifugation, the supernatant was allowed to react with thiobarbituric acid to produce the chromogen, thiobarbituric acid-malondialdehyde (TBA-MDA). Absorbance was measured at 440, 532, and 600 nm by using a micro-plate reader (Synergy Mx, Biotek Instruments Inc., Vermont, VT, USA). Protein oxidation (ProtOx) was estimated through measuring the protein carbonyl content, utilizing dinitrophenylhydrazine (DNPH) derivatization as described by Levine *et al.* (1994). Total non-enzymatic antioxidant capacity (TAC) was assayed by homogenizing and extracting 50 mg FW of tissue in 80% ethanol (v/v). After centrifugation, FRAP reagent (0.3 M acetate buffer (pH 3.6), 0.01 mM TPTZ in 0.04 mMHCl and 0.02 M FeCl3·6H2O) was mixed with the extract and measured at 600 nm using a microplate reader (Benzie and Strain 1999). Trolox (0 to 650 μM) was used as standard. MDA was and expressed in nmol(MDA)·g^{-1} of FW. TAC was expressed in μmol(trolox)·g^{-1} of FW.

Antioxidant enzymes activity

Soluble protein was extracted according to Murshed *et al.* (2008) and quantified by the Lowry method (Lowry et al. 1951). All enzyme activities were determined in 200 µL volume kinetic reactions at 25°C, using a micro-plate reader (Synergy Mx, Biotek Instruments Inc., Vermont, USA). Ascorbate peroxidase (APX) and dehydroascorbate reductase (DHAR) activities were measured by the method of Murshed *et al.* (2008) using 0.05 M MES/KOH) buffer (pH 6.0). Superoxide dismutase (SOD) activity was determined according to Dhindsa, Plumb-Dhindsa and Reid (1982) by measuring the inhibition of NBT reduction at 560 nm. Catalase (CAT) activity was assayed by monitoring the decomposition rate of H_2O_2 at 240 nm (Aebi 1984). Activities were expressed as µmol(AsA)·mg(protein)$^{-1}$·min^{-1} for APX and DHAR, µmol(H_2O_2)·mg(protein)$^{-1}$·min^{-1} for CAT and µmol(SOD)·mg(protein)$^{-1}$·min^{-1} for SOD.

References

Aebi H (1984) [13] Catalase in vitro. Methods Enzymol 105: 121–126

Bentsink L, Alonso-Blanco C, Vreugdenhil D, et al (2000) Genetic analysis of seed-soluble oligosaccharides in relation to seed storability of Arabidopsis. Plant Physiol 124:1595–1604. https://doi.org/10.1104/pp.124.4.1595

Benzie IFF, Strain JJ (1999) Ferric reducing/antioxidant power assay: Direct measure of total antioxidant activity of biological fluids and modified version for simultaneous measurement of total antioxidant power and ascorbic acid concentration. Methods Enzymol 299:15–27. https://doi.org/10.1016/S0076-6879(99)99005-5

Carreno-Quintero N, Undas A, Bachem CWB, et al (2014) Cross-platform comparative analyses of genetic variation in amino acid content in potato tubers. Metabolomics 10:1239–1257. https://doi.org/10.1007/s11306-014-0661-y

Dhindsa RS, Plumb-Dhindsa PL, Reid DM (1982) Leaf senescence and lipid peroxidation: Effects of some phytohormones, and scavengers of free radicals and singlet oxygen. Physiol Plant 56:453–457. https://doi.org/10.1111/j.1399-3054.1982.tb04539.x

He H, De Souza Vidigal D, Basten Snoek L, et al (2014) Interaction between parental environment and genotype affects plant and seed performance in Arabidopsis. J Exp Bot 65:6603–6615. https://doi.org/10.1093/jxb/eru378

Hodges DM, DeLong JM, Forney CF, Prange RK (1999) Improving the thiobarbituric acid-reactive-substances assay for estimating lipid peroxidation in plant tissues containing anthocyanin and other interfering compounds. Planta 207:604–611. https://doi.org/10.1007/s004250050524

Levine RL, Williams JA, Stadtman EP, Shacter E (1994) [37] Carbonyl assays for determination of oxidatively modified proteins. Methods Enzymol 233: 346–357

Lowry OH, Rosebrough NJ, Farr AL, Randall RJ (1951) Protein measurement with the Folin-Phenol Reagent. J Biol Chemistry 193: 265-275

Murshed R, Lopez-Lauri F, Sallanon H (2008) Microplate quantification of enzymes of the plant ascorbate-glutathione cycle. Anal Biochem 383:320–322. https://doi.org/10.1016/j.ab.2008.07.020
