# Supplementary material for: Drought tolerance in selected aerobic and upland rice varieties is driven by different metabolic and antioxidative responses
Source: Planta. 2021 Jun 25;254(1):13. doi: 10.1007/s00425-021-03659-4 (PMC8233253; doi:10.1007/s00425-021-03659-4)
Supplement: Supplementary file 3 — Supplementary file3 (PDF 3733 KB) [file 425_2021_3659_MOESM3_ESM.pdf]

Table S1. Dry weight (DW) of leaves, stems, roots, and total biomass, leaf relative water content (RWC) and the number of tillers of the three varieties under control and drought conditions at the four time points of sampling. Each value represents the mean ± standard deviation (SD) of three biological replicates. ***, **, * are the t-test's *P*-values of significance between control and drought replicates with $P < 0.001$, $0.001 < P < 0.01$, $0.01 < P < 0.05$, respectively.

		LEAVES DW				STEMS DW				ROOTS DW			
**Genotype**	**Time point**	**Control Mean ± SD**	**Drought Mean ± SD**	**t-test *P*-value**	**t-test sign.**	**Control Mean ± SD**	**Drought Mean ± SD**	**t-test *P*-value**	**t-test sign.**	**Control Mean ± SD**	**Drought Mean ± SD**	**t-test *P*-value**	**t-test sign.**
IR64	1	1.325±0.114	1.056±0.100	0.038	*	1.376±0.146	1.103±0.159	0.095		0.66±0.0190	0.707±0.124	0.581	
	2	1.711±0.054	1.369±0.031	0.002	**	1.812±0.005	1.522±0.040	0.006	**	0.812±0.093	0.882±0.091	0.400	
	3	2.584±0.167	1.423±0.067	0.003	**	2.996±0.254	1.947±0.084	0.012	*	1.467±0.128	1.260±0.054	0.091	
	4	3.474±0.219	0.777±0.155	0.000	***	4.471±0.336	1.894±0.257	0.000	***	2.571±0.525	1.158±0.076	0.041	*
Apo	1	0.878±0.070	0.851±0.067	0.651		1.055±0.095	1.044±0.072	0.878		0.454±0.067	0.468±0.050	0.783	
	2	1.510±0.128	1.172±0.087	0.024	*	1.744±0.132	1.573±0.191	0.280		0.704±0.062	0.855±0.169	0.258	
	3	2.028±0.182	1.268±0.125	0.006	**	2.505±0.325	1.796±0.227	0.042	*	1.205±0.420	0.985±0.130	0.464	
	4	2.777±0.225	1.382±0.016	0.008	**	3.668±0.165	2.106±0.069	0.001	**	1.638±0.100	1.047±0.068	0.002	**
Ri-7	1	0.869±0.125	1.024±0.089	0.163		0.978±0.142	1.103±0.100	0.288		0.472±0.123	0.621±0.104	0.186	
	2	1.207±0.189	1.227±0.120	0.882		1.481±0.193	1.745±0.144	0.137		0.888±0.091	0.944±0.094	0.495	
	3	1.812±0.165	1.187±0.158	0.009	**	2.057±0.187	1.871±0.024	0.225		1.315±0.094	1.178±0.040	0.112	
	4	2.548±0.113	1.059±0.069	0.000	***	2.993±0.113	2.106±0.094	0.000	***	1.747±0.109	1.142±0.194	0.054	*

		TOTAL DW				LEAF RWC				NUMBER OF TILLERS			
**Genotype**	**Time point**	**Control Mean ± SD**	**Drought Mean ± SD**	**t-test *P*-value**	**t-test sign.**	**Control Mean ± SD**	**Drought Mean ± SD**	**t-test *P*-value**	**t-test sign.**	**Control Mean ± SD**	**Drought Mean ± SD**	**t-test *P*-value**	**t-test sign.**
IR64	1	3.360±0.278	2.866±0.351	0.132		94.559±3.093	95.013±0.504	0.824		13.000±1.000	12.667±0.577	0.649	
	2	4.335±0.091	3.773±0.094	0.002	**	92.448±0.494	92.634±1.971	0.887		16.333±1.528	13.000±1.000	0.042	*
	3	7.048±0.349	4.629±0.146	0.003	**	96.300±1.101	24.058±4.691	0.000	***	20.333±2.517	14.000±1.000	0.035	*
	4	10.516±0.633	3.829±0.375	0.000	***	92.772±1.963	93.771±2.660	0.631		21.333±0.577	15.000±1.000	0.002	**
Apo	1	2.387±0.230	2.363±0.187	0.894		94.770±3.114	95.149±2.277	0.874		7.667±0.577	7.333±0.577	0.519	
	2	3.959±0.306	3.600±0.429	0.310		92.854±1.326	91.096±2.023	0.287		9.000±1.000	8.333±0.577	0.387	
	3	5.738±0.919	4.049±0.472	0.066		93.765±2.767	65.571±6.019	0.006	**	8.667±0.577	7.333±1.155	0.173	
	4	8.084±0.487	4.536±0.017	0.006	**	92.811±0.833	93.234±1.191	0.644		9.667±1.528	8.667±0.577	0.379	
Ri-7	1	2.319±0.362	2.748±0.282	0.185		92.236±0.518	89.117±1.649	0.071		5.667±0.577	5.667±0.577	1.000	
	2	3.575±0.462	3.917±0.276	0.346		89.934±3.431	89.930±1.761	0.999		7.333±2.082	7.000±0.000	0.808	
	3	5.184±0.439	4.236±0.095	0.059		95.992±0.742	73.082±2.815	0.003	**	7.333±0.577	6.667±0.577	0.230	
	4	7.289±0.308	4.307±0.238	0.002	**	95.975±0.779	92.575±1.636	0.051		9.000±1.000	7.333±1.155	0.133	

Table S2. Levels of the 39 metabolites, nutrient ions and oxidative stress markers/enzymes measured in control leaf samples of the three varieties at each time point (TP1, TP2, TP3, TP4). Each value represents the mean and standard deviation (St.Dev) of four biological replicates (for a total of 12 plants).

			TP1						TP2						TP3						TP4					
			IR64		Apo		Ri-7		IR64		Apo		Ri-7		IR64		Apo		Ri-7		IR64		Apo		Ri-7	
			Mean	St.Dev	Mean	St.Dev	Mean	St.Dev	Mean	St.Dev	Mean	St.Dev	Mean	St.Dev	Mean	St.Dev	Mean	St.Dev	Mean	St.Dev	Mean	St.Dev	Mean	St.Dev	Mean	St.Dev	Mean	St.Dev
BCAAs	Leu	pmol µL-1 g-1 DW	34.63	1.80	36.60	1.84	45.00	8.10	79.71	8.58	33.35	2.64	46.66	7.79	46.18	8.57	33.29	3.62	32.25	1.14	58.77	4.86	46.28	2.72	43.88	3.79
	Ile	pmol µL-1 g-1 DW	35.61	1.31	38.61	2.34	43.25	6.65	68.01	8.44	33.70	2.60	48.79	8.03	41.55	5.94	33.42	2.44	35.23	1.48	54.06	5.01	44.80	1.60	47.02	3.52
	Val	pmol µL-1 g-1 DW	52.13	2.13	78.78	9.00	58.53	7.62	93.52	10.85	55.53	2.61	70.44	11.03	49.99	5.47	50.07	1.46	50.50	4.02	68.65	4.94	54.08	0.63	60.22	2.86
AAAs	Phe	pmol µL-1 g-1 DW	46.69	3.41	47.45	4.09	55.43	6.62	77.19	9.00	46.00	1.64	49.06	4.38	52.03	4.98	45.95	0.74	40.61	1.88	64.70	6.32	55.21	4.47	46.55	4.88
	Trp	pmol µL-1 g-1 DW	20.45	0.94	21.51	1.82	21.01	1.52	27.23	1.63	18.36	1.53	20.77	1.78	22.03	1.38	18.14	0.44	17.78	0.48	22.87	0.72	20.81	0.70	21.38	1.12
	Tyr	pmol µL-1 g-1 DW	45.54	4.95	44.70	6.73	46.73	5.10	59.20	4.19	35.76	1.00	43.92	3.89	39.57	2.34	35.75	1.24	36.25	2.25	45.33	3.03	38.23	2.11	40.60	4.04
Stress responsive AAs	Orn	pmol µL-1 g-1 DW	20.58	1.88	24.34	3.08	18.11	1.08	18.88	0.95	20.70	2.18	18.38	1.44	19.03	1.07	22.02	0.75	19.70	0.96	18.16	0.46	19.59	1.60	19.50	0.73
	Pro	pmol µL-1 g-1 DW	59.74	6.13	65.44	6.88	60.28	3.74	82.14	4.17	62.14	1.61	52.42	3.27	60.51	3.05	58.56	1.35	50.14	1.84	59.10	2.08	58.04	2.68	48.98	2.57
	GABA	pmol µL-1 g-1 DW	27.70	11.27	36.30	7.63	6.91	2.54	35.62	6.02	28.23	4.43	27.77	4.48	12.43	5.97	24.11	2.88	22.91	6.00	14.74	8.45	10.51	3.02	6.56	2.11
Photorespiratory AAs	Gly	pmol µL-1 g-1 DW	137.45	6.27	523.31	127.49	177.19	50.53	131.12	22.41	245.52	52.69	98.51	11.02	89.18	10.19	259.79	37.24	167.62	44.79	118.15	17.82	151.83	26.83	92.70	20.68
	Ser	pmol µL-1 g-1 DW	849.90	39.00	929.10	64.06	374.80	52.05	592.10	45.26	811.78	150.40	326.40	23.69	477.28	74.71	530.28	141.65	316.55	44.66	353.58	22.81	247.08	28.31	161.13	15.41
N remobilisation AAs	Gln	pmol µL-1 g-1 DW	212.58	44.77	341.99	44.74	106.46	38.95	121.18	26.00	260.44	76.56	188.51	62.11	84.00	29.20	153.86	73.34	298.07	76.33	37.25	5.69	25.97	3.17	24.62	4.88
	Glu	pmol µL-1 g-1 DW	1170.63	47.39	1539.70	75.05	1239.12	67.09	1089.72	61.98	1619.42	137.95	1088.08	48.68	852.22	68.04	1303.12	113.22	976.33	129.17	740.99	21.57	912.06	47.35	563.61	17.89
	Asn	pmol µL-1 g-1 DW	98.20	14.05	95.36	18.94	23.77	3.86	58.70	6.89	72.44	19.65	25.55	1.25	39.13	8.99	32.52	6.45	26.55	7.88	26.38	5.38	15.64	0.79	15.63	1.03
	Asp	pmol µL-1 g-1 DW	339.53	53.03	415.05	33.02	304.97	58.27	232.91	28.87	360.08	55.89	329.54	30.61	226.42	55.45	284.54	63.91	325.94	57.37	104.55	9.04	107.10	5.52	98.68	11.74
Other AAs	Ala	pmol µL-1 g-1 DW	452.78	43.95	633.90	64.72	240.65	39.51	369.98	34.82	462.05	31.78	394.25	40.82	309.98	52.63	380.08	61.16	403.30	35.29	220.95	18.27	196.45	11.68	177.30	14.51
	Thr	pmol µL-1 g-1 DW	245.35	27.84	299.80	20.09	132.70	9.21	161.17	9.53	251.29	39.99	102.96	8.62	152.09	16.31	195.59	24.10	83.55	12.14	104.78	2.85	107.48	5.18	66.14	2.76
	Met	pmol µL-1 g-1 DW	33.91	1.87	36.58	1.50	27.57	1.56	37.99	1.65	30.74	1.81	27.50	1.64	32.38	2.09	28.05	0.89	26.12	1.32	30.56	1.21	27.37	1.51	28.39	1.30
	His	pmol µL-1 g-1 DW	28.85	2.11	30.35	1.31	28.11	1.33	36.25	5.82	27.68	2.76	29.81	2.99	31.43	3.66	27.85	1.68	27.69	1.64	29.24	2.37	29.78	3.83	30.39	1.64
	Arg	pmol µL-1 g-1 DW	53.69	5.95	83.13	9.22	45.37	1.63	49.56	4.95	57.97	5.65	42.86	1.96	49.79	1.80	64.98	6.84	43.42	0.90	44.90	3.25	47.14	2.86	43.75	1.06
	Lys	pmol µL-1 g-1 DW	36.50	4.70	35.14	2.24	30.37	1.38	46.24	3.80	32.15	2.91	32.57	3.15	35.78	3.21	33.00	1.16	27.57	2.73	37.00	3.84	33.58	0.83	37.74	1.42
Sugars	Sucrose	mg L-1 g-1 DW	8.22	0.24	7.60	0.62	8.35	0.80	8.30	0.25	8.28	0.45	8.82	0.54	8.58	0.46	8.76	0.25	8.61	0.58	10.84	0.24	10.99	0.42	8.18	0.31
	Glucose	mg L-1 g-1 DW	0.06	0.01	0.07	0.02	0.09	0.04	0.12	0.04	0.06	0.01	0.12	0.02	0.06	0.00	0.06	0.01	0.10	0.01	0.10	0.01	0.08	0.00	0.09	0.01
	Fructose	mg L-1 g-1 DW	0.07	0.02	0.06	0.03	0.10	0.05	0.11	0.04	0.05	0.01	0.15	0.06	0.05	0.00	0.05	0.02	0.14	0.03	0.09	0.04	0.11	0.03	0.10	0.05
	Trehalose	mg L-1 g-1 DW	0.00	0.00	0.00	0.00	0.00	0.00	0.01	0.00	0.00	0.00	0.00	0.00	0.01	0.00	0.00	0.00	0.01	0.00	0.01	0.00	0.00	0.00	0.01	0.00
	Xylose	mg L-1 g-1 DW	0.04	0.01	0.04	0.01	0.02	0.01	0.03	0.01	0.04	0.01	0.01	0.01	0.03	0.01	0.02	0.00	0.01	0.01	0.02	0.02	0.02	0.01	0.01	0.02
Organic acids	Cit	mg L-1 g-1 DW	0.94	0.07	1.19	0.42	1.44	0.17	0.89	0.07	1.12	0.06	1.81	0.28	1.11	0.19	1.43	0.14	1.44	0.25	1.07	0.07	1.17	0.17	1.67	0.10
	Isocit	mg L-1 g-1 DW	0.72	0.02	0.77	0.17	0.53	0.13	0.61	0.09	0.86	0.11	0.66	0.16	1.05	0.24	1.18	0.22	0.74	0.19	0.99	0.21	1.01	0.12	0.65	0.08
	alpha-ket	mg L-1 g-1 DW	0.67	0.22	0.23	0.12	0.26	0.11	0.55	0.19	0.53	0.25	0.38	0.23	0.53	0.26	0.45	0.21	0.41	0.13	0.81	0.03	0.87	0.10	0.85	0.06
Nutrient ions	Sulfate	mg L-1 g-1 DW	5.92	0.48	4.50	0.44	6.70	1.08	6.66	1.57	4.68	0.62	9.27	1.67	5.70	0.36	6.58	1.18	9.43	1.62	5.24	0.41	6.41	0.44	8.95	0.90
	Nitrate	mg L-1 g-1 DW	1.95	0.48	2.76	0.64	0.80	0.59	0.52	0.52	1.28	0.31	0.17	0.04	0.82	0.63	0.47	0.22	0.40	0.45	0.78	0.98	0.50	0.48	0.14	0.27
	Phosphate	mg L-1 g-1 DW	1.73	0.31	1.85	0.06	1.69	0.14	2.18	0.36	1.75	0.11	2.26	0.22	2.34	0.49	1.94	0.12	2.06	0.24	2.45	0.30	1.98	0.22	2.92	0.48
Oxidative stress marker	TAC	µmol trolox g^{-1} FW	26.99	8.69	27.56	15.08	26.61	3.63	23.48	6.32	26.95	8.19	22.79	4.11	19.15	1.25	20.23	4.66	18.98	3.46	17.04	2.55	14.54	0.90	17.46	2.67
	ProtOx	mg carbonyl mg^{-1} protein	1.22	0.15	1.20	0.11	1.13	0.11	1.24	0.18	1.26	0.27	1.31	0.13	2.03	0.35	1.38	0.19	1.35	0.11	1.39	0.32	1.20	0.11	1.22	0.10
	MDA	nmol(MDA) g^{-1} FW	82.05	10.28	60.33	10.88	73.92	12.75	72.22	6.95	74.30	15.35	60.65	15.03	95.35	23.14	155.21	112.66	72.54	13.87	58.50	9.60	64.85	10.40	61.96	6.08
Oxidative stress enzyme	DHAR	umol(AsA) $mg(protein)^{-1}$ min^{-1}	0.25	0.02	0.25	0.03	0.29	0.05	0.21	0.03	0.21	0.04	0.22	0.02	0.22	0.03	0.17	0.04	0.21	0.05	0.29	0.09	0.21	0.07	0.26	0.02
	APX	umol(AsA) $mg(protein)^{-1}$ min^{-1}	0.61	0.11	0.55	0.03	0.66	0.16	0.43	0.29	0.39	0.36	0.31	0.01	0.52	0.03	0.51	0.13	0.69	0.12	0.81	0.12	0.73	0.04	0.68	0.24
	CAT	umol(H_2O_2) $mg(protein)^{-1}$ min^{-1}	28.02	5.51	26.36	1.44	19.08	13.43	16.85	1.40	21.13	4.63	6.02	4.74	11.36	1.16	15.29	1.06	22.93	2.39	24.60	10.23	24.67	3.70	16.63	9.06
	SOD	unit(SOD) $mg(protein)^{-1}$ min^{-1}	198.91	21.34	190.52	45.58	269.82	25.34	261.99	20.15	223.86	28.60	245.74	44.43	252.24	53.34	181.31	109.74	255.53	47.91	264.37	83.77	248.09	17.25	267.06	22.90

Table S3. Levels of the 39 metabolites, nutrient ions and oxidative stress markers/enzymes measured in drought stressed leaf samples of the three varieties at each time point (TP1, TP2, TP3, TP4). Each value represents the mean and standard deviation (St.Dev) of four biological replicates (for a total of 12 plants).

			TP1						TP2						TP3						TP4					
			IR64		Apo		Ri-7		IR64		Apo		Ri-7		IR64		Apo		Ri-7		IR64		Apo		Ri-7	
			Mean	St.Dev	Mean	St.Dev	Mean	St.Dev	Mean	St.Dev	Mean	St.Dev	Mean	St.Dev	Mean	St.Dev	Mean	St.Dev	Mean	St.Dev	Mean	St.Dev	Mean	St.Dev	Mean	St.Dev
BCAAs	Leu	pmol μL-1 g-1 DW	42.04	3.59	32.73	0.93	59.95	7.70	93.75	5.24	61.02	8.58	56.99	5.18	505.74	45.21	294.81	58.01	160.12	39.94	57.36	4.98	38.72	3.14	40.43	3.65
	Ile	pmol μL-1 g-1 DW	42.80	3.34	34.23	1.20	60.96	6.81	89.35	3.13	57.57	8.95	57.09	3.08	799.32	92.21	416.05	84.76	170.05	46.98	60.98	6.72	46.07	4.57	46.09	3.39
	Val	pmol μL-1 g-1 DW	53.30	4.89	49.81	2.87	82.83	8.54	117.85	6.81	75.39	15.37	85.68	4.49	1520.98	200.15	858.84	188.18	296.90	84.63	148.23	19.87	95.66	18.67	93.28	9.65
AAAs	Phe	pmol μL-1 g-1 DW	54.30	3.40	48.45	2.91	60.02	10.15	90.87	7.42	61.41	5.28	49.52	6.57	1052.35	175.48	535.65	130.88	178.35	70.99	77.98	14.06	58.41	11.82	44.65	2.75
	Trp	pmol μL-1 g-1 DW	21.85	1.48	20.67	1.63	30.69	6.14	44.24	7.54	29.06	6.33	30.97	3.71	730.85	112.43	539.47	129.86	209.79	100.94	103.20	36.58	156.79	23.14	23.82	3.01
	Tyr	pmol μL-1 g-1 DW	47.37	2.96	43.70	3.93	49.53	5.36	60.76	4.34	48.07	1.06	41.67	5.05	295.51	50.47	185.05	40.90	93.02	30.39	61.18	5.38	45.35	5.14	46.36	3.14
Stress responsive AAs	Orn	pmol μL-1 g-1 DW	19.93	0.88	20.73	0.97	19.34	0.66	19.03	1.38	18.58	0.84	18.06	0.56	44.47	3.02	25.07	1.48	18.75	0.46	98.64	7.34	170.74	19.71	181.82	9.71
	Pro	pmol μL-1 g-1 DW	58.61	2.44	55.73	1.85	56.10	7.50	95.52	14.76	65.06	8.15	54.90	6.59	5899.88	821.84	3219.15	479.74	773.43	464.61	491.85	151.20	222.21	114.37	61.49	4.76
	GABA	pmol μL-1 g-1 DW	29.73	8.31	25.04	9.67	9.28	3.27	26.27	3.78	24.04	7.48	31.46	7.21	1431.11	229.42	147.24	38.15	85.27	22.16	155.82	25.25	75.37	19.19	29.06	2.53
Photorespiratory AAs	Gly	pmol μL-1 g-1 DW	130.61	21.95	241.96	74.20	127.70	12.21	127.90	17.44	132.95	48.26	69.91	10.01	358.77	38.85	387.49	61.39	113.07	35.69	705.08	78.14	675.57	219.79	511.71	80.56
	Ser	pmol μL-1 g-1 DW	728.28	67.05	850.73	62.77	272.93	48.37	468.78	81.58	392.25	61.39	165.98	16.74	789.80	104.81	880.65	101.38	527.33	129.03	903.48	79.95	550.30	58.61	373.10	20.80
N remobilisation AAs	Gln	pmol μL-1 g-1 DW	186.63	28.48	274.21	29.70	102.42	56.25	141.21	45.52	87.49	53.49	102.85	7.44	4066.71	773.75	2797.52	651.27	992.78	335.50	1208.00	165.34	939.93	177.28	409.64	47.69
	Glu	pmol μL-1 g-1 DW	1192.50	34.28	1555.74	49.43	1142.47	89.02	1383.34	151.91	1265.94	140.59	820.07	59.85	1223.24	109.98	1752.31	127.94	1413.10	113.34	1242.68	49.61	1112.10	55.97	867.62	46.76
	Asn	pmol μL-1 g-1 DW	61.06	8.21	44.51	7.54	22.03	4.79	37.27	4.76	24.84	4.05	21.34	0.67	1556.52	216.09	1488.47	273.77	833.97	314.01	1299.24	216.31	520.05	104.09	73.02	15.04
	Asp	pmol μL-1 g-1 DW	352.07	30.16	450.39	20.00	196.18	21.11	236.19	42.69	222.29	38.20	151.64	7.39	492.52	34.35	394.79	40.46	280.97	19.38	461.99	28.90	409.65	13.46	287.61	30.21
Other AAs	Ala	pmol μL-1 g-1 DW	396.10	16.91	414.60	31.36	191.15	33.78	303.08	14.55	295.55	24.47	311.33	31.78	1566.05	296.05	805.15	36.74	549.53	61.59	680.48	48.97	670.65	106.06	531.30	70.29
	Thr	pmol μL-1 g-1 DW	174.37	2.77	246.41	8.90	112.48	14.18	153.62	15.32	145.01	13.46	85.01	5.02	1159.87	141.56	778.35	160.18	251.15	76.54	431.16	56.13	485.29	5.02	283.00	10.82
	Met	pmol μL-1 g-1 DW	30.00	2.61	29.88	0.30	27.19	0.90	34.85	1.29	29.21	1.54	26.32	1.33	154.28	21.68	114.26	20.42	49.29	12.26	54.22	5.17	35.37	3.76	34.13	2.04
	His	pmol μL-1 g-1 DW	30.62	5.53	28.89	1.81	34.32	5.42	44.54	9.85	30.96	1.95	32.54	4.93	514.31	90.09	371.61	104.53	131.81	77.14	76.29	42.60	119.47	42.67	32.89	4.72
	Arg	pmol μL-1 g-1 DW	48.20	4.14	60.06	5.54	43.54	2.22	53.36	6.28	44.16	4.00	40.81	1.00	535.90	131.76	283.20	112.98	93.95	20.82	375.17	110.93	552.16	24.89	325.80	27.77
	Lys	pmol μL-1 g-1 DW	36.41	3.14	31.34	2.44	33.71	1.75	55.43	2.09	38.15	5.38	36.56	1.92	413.28	62.86	223.78	65.93	55.31	4.03	167.88	45.14	179.13	11.30	94.85	9.78
Sugars	Sucrose	mg L-1 g-1 DW	9.03	0.40	8.37	0.30	9.78	0.89	8.58	0.57	8.85	0.22	8.49	0.46	6.99	0.53	6.67	0.60	6.88	0.34	8.54	0.40	10.71	0.45	8.38	0.35
	Glucose	mg L-1 g-1 DW	0.12	0.01	0.07	0.01	0.37	0.09	1.36	0.40	0.59	0.26	0.85	0.13	1.05	0.17	1.45	0.20	1.79	0.10	0.36	0.10	0.46	0.16	0.08	0.01
	Fructose	mg L-1 g-1 DW	0.08	0.02	0.08	0.03	0.43	0.10	1.05	0.33	0.57	0.22	0.87	0.11	0.54	0.11	1.34	0.42	1.64	0.08	0.38	0.13	0.64	0.18	0.06	0.02
	Trehalose	mg L-1 g-1 DW	0.01	0.00	0.00	0.00	0.01	0.00	0.01	0.00	0.00	0.00	0.01	0.00	0.02	0.00	0.02	0.00	0.01	0.00	0.01	0.00	0.02	0.00	0.02	0.00
	Xylose	mg L-1 g-1 DW	0.02	0.01	0.04	0.01	0.03	0.01	0.10	0.06	0.05	0.03	0.06	0.04	0.19	0.01	0.17	0.02	0.13	0.05	0.07	0.01	0.08	0.01	0.03	0.01
Organic acids	Cit	mg L-1 g-1 DW	1.03	0.12	1.72	0.27	1.47	0.42	0.98	0.22	1.15	0.14	1.21	0.14	0.49	0.05	0.50	0.03	0.67	0.01	1.54	0.23	1.64	0.17	2.07	0.17
	Isocit	mg L-1 g-1 DW	0.84	0.08	0.97	0.10	0.53	0.10	1.14	0.41	0.86	0.18	0.69	0.07	0.09	0.02	0.34	0.10	0.41	0.03	0.36	0.03	0.32	0.09	0.33	0.05
	alpha-ket	mg L-1 g-1 DW	0.50	0.26	0.73	0.10	0.69	0.41	0.96	0.11	0.82	0.09	0.92	0.24	0.25	0.17	0.46	0.09	0.51	0.08	0.51	0.24	0.64	0.23	0.63	0.06
Nutrient ions	Sulfate	mg L-1 g-1 DW	8.31	1.29	4.26	0.58	8.40	1.26	7.75	2.86	5.44	1.09	15.25	1.40	9.68	1.60	8.20	1.10	15.27	1.06	4.88	0.73	5.73	0.44	11.74	0.24
	Nitrate	mg L-1 g-1 DW	0.74	0.31	3.69	0.55	0.30	0.26	0.60	0.27	0.94	0.35	0.15	0.26	0.61	0.29	0.48	0.31	0.04	0.05	3.23	0.60	1.12	0.28	0.78	0.10
	Phosphate	mg L-1 g-1 DW	1.05	0.08	1.38	0.06	1.03	0.12	0.86	0.26	0.98	0.08	0.99	0.04	1.31	0.11	0.77	0.05	1.19	0.13	2.20	0.35	1.52	0.12	1.23	0.14
Oxidative stress marker	TAC	μmol trolox g^{-1} FW	36.09	19.15	29.84	12.41	27.25	13.45	24.64	4.70	30.64	10.34	31.54	9.93	17.50	5.79	45.57	17.12	45.03	21.03	20.82	9.89	21.31	8.07	20.24	1.46
	ProtOx	mg carbonyl mg^{-1} protein	1.22	0.15	1.20	0.11	1.13	0.11	1.24	0.18	1.26	0.27	1.31	0.13	2.03	0.35	1.38	0.19	1.35	0.11	1.39	0.32	1.20	0.11	1.22	0.10
	MDA	nmol(MDA) g^{-1} FW	63.88	11.42	63.71	12.65	74.09	14.62	120.39	31.16	134.13	91.21	144.61	94.35	96.04	44.58	246.44	118.37	210.45	163.46	73.98	28.20	146.97	95.50	122.43	69.55
Oxidative stress enzyme	DHAR	umol(AsA) mg(protein)$^{-1}$ min^{-1}	0.22	0.02	0.28	0.07	0.23	0.05	0.23	0.02	0.18	0.02	0.21	0.06	0.26	0.02	0.44	0.06	0.34	0.04	0.30	0.04	0.24	0.08	0.17	0.02
	APX	umol(AsA) mg(protein)$^{-1}$ min^{-1}	0.66	0.03	0.70	0.13	0.49	0.11	0.41	0.17	0.46	0.19	0.40	0.16	0.62	0.07	0.75	0.09	0.97	0.55	0.67	0.13	0.71	0.09	0.74	0.08
	CAT	umol(H_2O_2) mg(protein)$^{-1}$ min^{-1}	18.30	1.15	23.27	6.30	13.22	8.61	13.58	3.09	22.09	1.30	14.88	1.67	19.23	2.42	23.11	0.37	19.62	4.50	22.43	1.31	23.45	7.97	22.54	2.96
	SOD	unit(SOD) mg(protein)$^{-1}$ min^{-1}	207.70	43.17	220.17	41.02	244.41	96.59	218.26	22.29	194.63	43.74	262.66	24.06	301.99	14.55	357.40	9.98	343.25	100.88	228.86	9.08	203.80	62.55	205.21	20.93

Table S4. Bonferroni corrected *P*-values of the factors (Var: variety; Treat: treatment; Int: interaction) of the two-way ANOVA for the 39 metabolic and oxidative stress markers/enzymes at each time point.

	TP1			TP2			TP3			TP4		
**Variable**	**Treat**	**Var**	**Int**	**Treat**	**Var**	**Int**	**Treat**	**Var**	**Int**	**Treat**	**Var**	**Int**
Leu	0.323732	4.18E-05	0.105931	9.29E-05	1.36E-07	0.108519	1.00E-15	2.81E-06	0.006779	0.395695	8.01E-06	1
Ile	0.061054	4.89E-05	0.008878	7.62E-05	1.40E-06	0.667131	9.97E-17	4.07E-07	6.51E-06	1	0.001912	1
Val	1	0.003825	2.91E-05	0.004022	3.27E-05	1	2.11E-17	1.33E-06	1.07E-06	1.02E-08	0.000799	1
Phe	1	0.299896	1	0.031692	6.74E-08	0.967753	4.85E-14	3.32E-06	0.000148	1	0.001045	1
Trp	0.24312	0.135596	0.111625	5.47E-06	0.000588	1	2.51E-15	0.000999	0.017369	8.25E-11	2.79E-07	2.83E-07
Tyr	1	1	1	0.323832	3.32E-07	0.015616	2.24E-12	0.000242	0.00134	0.00066	0.002156	1
Orn	1	0.024391	1	1	1	1	4.76E-10	5.73E-10	5.42E-11	7.08E-22	5.72E-07	2.35E-05
Pro	1	1	1	1	2.07E-06	1	1.33E-16	3.74E-06	5.10E-05	1.15E-07	2.50E-05	0.000341
GABA	1	8.80E-06	1	1	1	1	1.19E-13	2.12E-05	5.67E-09	1.17E-09	0.000301	1
Gly	0.013758	2.66E-06	0.395954	0.029561	0.000122	0.951164	0.000803	1.12E-05	6.98E-06	1.07E-11	0.274528	1
Ser	0.017931	1.02E-11	1	6.77E-07	8.69E-09	0.206464	0.000139	0.003594	1	3.46E-13	1.00E-10	1
Gln	1	2.02E-05	1	0.031735	1	0.13313	1.96E-11	1	8.25E-05	3.76E-19	5.02E-06	0.01297
Glu	1	6.85E-08	1	0.389993	1.92E-06	0.000355	9.75E-07	1.11E-05	1	1.18E-12	1.08E-10	0.000107
Asn	0.00034	2.28E-09	0.16731	3.36E-06	5.53E-06	0.004916	1.98E-16	0.050806	1	3.54E-18	1.20E-11	1.84E-09
Asp	1	3.32E-06	0.028262	3.78E-05	0.40715	0.004556	0.00339	1	0.003204	3.10E-17	0.000279	0.010804
Ala	0.001648	1.54E-09	1	4.28E-06	1	0.719631	1.30E-10	0.003416	2.14E-06	2.03E-14	0.030763	1
Thr	5.20E-05	1.71E-11	1	0.000258	5.35E-09	0.0166	5.92E-12	3.25E-08	0.009317	2.32E-20	1.13E-10	1
Met	0.002199	0.000101	0.30839	0.380511	9.93E-08	1	2.48E-12	2.24E-06	0.000244	3.87E-08	1.47E-05	0.003151
His	1	1	1	1	0.03738	1	3.23E-12	0.002151	0.00925	0.000134	0.064667	0.04164
Arg	0.009646	2.98E-07	0.369434	1	0.007817	0.100533	6.22E-11	7.50E-06	0.000134	6.82E-18	0.015444	0.185711
Lys	1	0.700675	1	0.016406	2.23E-06	1	8.17E-15	8.42E-10	6.49E-08	1.45E-14	0.015562	0.00074
Suc	0.025944	0.319962	1	1	1	1	1.71E-06	1	1	0.003505	9.91E-09	0.000215
Gluc	1.76E-05	4.81E-05	0.002115	1.34E-11	0.004482	1	8.43E-21	4.29E-06	1	1.24E-07	5.37E-06	1.02E-05
Fru	0.014255	0.001273	0.126409	5.61E-11	0.004223	1	1.99E-13	9.50E-05	0.575473	0.001542	0.001182	0.004933
Tre	1	1	1	9.76E-05	5.60E-06	0.500107	1.50E-05	1	0.99263	0.000225	1	0.006244
Xyl	1	1	1	0.049122	1	1	7.86E-10	0.115919	1	0.000999	0.078437	1
Cit	1	0.086735	1	1	0.000273	0.134006	2.45E-11	0.021368	1	8.16E-05	0.000154	1
Isocit	1	0.000897	1	0.846888	1	0.781255	1.07E-09	0.00147	7.08E-05	8.28E-09	1	1
alpha-ket	1	1	1	0.051114	1	1	1	1	1	0.101613	1	1
Sulphate	0.342348	2.51E-05	1	0.242827	2.20E-05	1	6.77E-05	7.79E-05	1	1	1.40E-09	0.040536
Nitrate	1	0.007575	1	1	0.002945	1	1	0.042994	0.965632	0.050509	1	1
Phosphate	1.37E-07	0.057731	1	3.34E-09	1	1	6.83E-09	0.004761	0.606972	2.34E-05	0.056322	0.002486
TAC	1	1	1	1	1	1	0.000366	1	1	1	1	1
ProtOx	1	1	1	1	1	1	0.005798	0.623198	0.310045	0.204756	1	1
MDA	1	1	1	0.286914	1	1	0.043082	1	1	0.495758	1	1
DHAR	1	1	1	1	1	1	1.16E-05	1	0.031677	1	1	1
APX	1	1	1	1	1	1	1	1	1	1	1	1
CAT	1	1	1	1	0.027727	0.262352	0.018386	0.004506	0.004263	1	1	1
SOD	1	1	1	1	1	1	0.151697	1	1	0.841559	1	1

Table S5. Levels of the 33 metabolites and oxidative stress markers/enzymes measured in control (CON) and drought stressed (DRO) leaf samples of the three varieties from the field experiment at the reproductive stage. Each value represents the mean of three plot replicates (for a total of 24 plants).

Metabolic or oxidative stress class			Field trial					
			IR64		Apo		Ri-7	
			CON	DRO	CON	DRO	CON	DRO
BCAAs	Leu	detctor response	13308.90	33522.93	12228.13	14484.27	12027.99	17599.97
	Ile	detctor response	22702.51	36705.07	21014.09	16914.63	19722.09	24742.42
	Val	detctor response	129632.54	122280.51	100998.92	72822.66	101137.06	98144.82
AAAs	Phe	detctor response	11135.33	30003.40	9090.20	11787.41	7984.95	12339.14
	Trp	detctor response	1396.41	3525.81	1475.79	1497.46	1255.97	1461.61
	Tyr	detctor response	25755.06	45851.75	18285.46	20751.69	16918.06	29315.16
Stress responsive AAs	Orn	detctor response	3177.92	6236.34	1489.19	2667.27	860.70	4161.34
	Pro	detctor response	104028.66	269285.44	84207.90	111237.56	77947.00	99036.00
Photorespiratory AAs	Gly	detctor response	122559.65	118415.44	68602.16	101884.49	81925.21	160023.18
	Ser	detctor response	486818.23	431372.97	217074.46	260968.96	182873.68	215269.91
N remobilisation AAs	Gln	detctor response	74061.94	90744.92	42400.36	50981.14	31208.70	71829.76
	Glu	detctor response	1068623.41	1295940.30	887022.97	964343.29	874891.73	844785.42
	Asn	detctor response	71633.05	69683.08	17491.89	16195.60	21889.15	18074.44
	Asp	detctor response	36805.34	51337.06	25547.85	26519.94	18070.08	18475.46
Other AAs	Ala	detctor response	869006.35	1228217.72	696449.92	1140252.81	691183.24	1190258.65
	Thr	detctor response	49849.09	59836.61	28380.30	28086.98	25894.09	23319.30
	Met	detctor response	4418.36	6470.62	3659.45	2854.87	3000.04	4914.87
	Arg	detctor response	1858.84	1020.86	817.93	916.32	788.61	1197.51
	Lys	detctor response	16968.50	27260.30	18664.06	14955.24	13832.40	12460.14
Sugars	Sucrose	A340 (absorbance)	0.531	0.488	0.255	0.358	0.458	0.367
	Glucose	A340 (absorbance)	0.068	0.081	0.070	0.086	0.057	0.088
	Fructose	A340 (absorbance)	0.089	0.100	0.087	0.112	0.078	0.099
	Trehalose	detctor response	13971.13	21373.21	16095.02	16540.87	14880.79	14567.14
Organic acids	Cit	detctor response	585032.99	728142.40	1041801.63	826640.12	1547728.15	735340.53
	Isocit	detctor response	183166.82	105397.84	153147.63	91207.78	177993.05	63893.17
	alpha-ket	detctor response	119644.91	144397.91	64910.05	56794.33	93191.49	80621.33
Oxidative stress marker	TAC	μmol trolox g^{-1} FW	5.082	7.226	7.339	4.615	7.500	4.361
	ProtOx	mg carbonyl mg^{-1} protein	0.523	0.741	0.481	1.099	0.653	1.076
	MDA	nmol(MDA) g^{-1} FW	13.823	31.620	24.351	22.733	13.840	32.222
Oxidative stress enzyme	DHAR	umol(AsA) $mg(protein)^{-1}$ min^{-1}	0.078	0.117	0.096	0.246	0.127	0.140
	APX	umol(AsA) $mg(protein)^{-1}$ min^{-1}	0.745	0.811	0.165	1.109	0.872	0.999
	CAT	umol(H_2O_2) $mg(protein)^{-1}$ min^{-1}	14.556	60.556	11.149	77.160	19.225	75.200
	SOD	unit(SOD) $mg(protein)^{-1}$ min^{-1}	116.609	380.323	83.195	490.002	71.812	693.934
